# Exploring an n-type conducting polymer (BBL) as a potential gas sensing material for NH_3_ and H_2_S detection

**DOI:** 10.1038/s41598-025-93977-4

**Published:** 2025-03-27

**Authors:** Sonu Sunny, Sushri Soumya Jena, Shivam Shah, Bhavika Gopalani, Arnab Hazra, Mohit Garg, Sarbani Ghosh

**Affiliations:** 1 Department of Chemical Engineering, Birla Institute of Technology and Science (BITS), Pilani Campus, Vidya Vihar, Pilani, 333031 Rajasthan India; 2 Department of Electrical and Electronics Engineering, Birla Institute of Technology and Science (BITS), Pilani Campus, Vidya Vihar, Pilani, 333031 Rajasthan India

**Keywords:** Chemical engineering, Materials for devices, Theory and computation

## Abstract

Conducting polymers (CPs) have garnered significant interest in being used as an active material in gas sensors mainly because of their structural flexibility, ease of synthesis, and enhanced performance at room temperature. The p-type CPs and their composites are mostly studied in gas sensing, which, unfortunately, exhibit limitations in terms of selectivity, stability, and sensitivity toward reducing gases. This study focuses on one of the widely studied n-type polymers, BBL(benzimidazobenzophenanthroline), as an active material for the detection of two reducing gases, namely, hydrogen sulfide (H$_{2}$S) and ammonia (NH$_{3}$), theoretically. Through molecular dynamics (MD) simulation and density functional theory (DFT) approach, we understand the adsorption behavior and selectivity of H$_{2}$S and NH$_{3}$ in the BBL film. The DFT calculated adsorption energy of the preferential site at the top of a $\pi -\pi$ stack for H$_{2}$S and NH$_{3}$ are – 0.22 eV and – 0.33 eV, respectively, and at the sides of a $\pi -\pi$ stack for H$_{2}$S and NH$_{3}$ are – 0.42 eV and – 0.47 eV, respectively. MD simulations show that adsorption takes place in the free voids within the thin films, and the overall structure of the polymer film remained almost unaltered upon gas adsorption without any apparent swelling or significant morphological changes in the film. Our results show that BBL displays remarkable adsorption along with a higher magnitude of charge transfer for ammonia over hydrogen sulfide gas and other common gases present in the air. Moreover, both H$_{2}$S and NH$_{3}$ gas adsorption happen without compromising the size of the $\pi -\pi$ stacked crystallites within the polymer film, which indicates, upon detection of reducing gases, the generated free electrons via the redox reactions between the gas molecules and polymer, will be able to be smoothly transported through the $\pi -\pi$ stack network present in the film. The detailed theoretical insights obtained from this study indicate the suitability of the n-type conducting polymer, BBL, for detecting reducing gases, NH$_{3}$ and H$_{2}$S.

## Introduction

Gas sensors are widely employed in our daily lives by detecting toxic and flammable gases that can cause harm to human health, e.g., CO$_{x}$(x = 1,2), NO$_{x}$(x = 1,2), NH$_{3}$, SO$_{x}$(x = 2,3) and H$_{2}$S, to detect fire, to detect odors in the home/office, to monitor environmental pollution in several process control industries, to monitor outdoor air quality, to detect food spoilage^1^, to name a few, overall ensuring environmental and human safety^2–4^. Finding an affordable and effective material to be used as an active layer of the gas sensor with high selectivity and sensitivity toward the analyte gas molecules, as well as with good stability and repeatability, is crucial to detecting harmful gas concentrations effectively.

Among the several toxic gases, we have considered two reducing gases, NH$_{3}$ and H$_{2}$S, which according to Occupational Safety and Health Administration (OSHA) and the Bureau of Labor Statistics, are considered an extremely dangerous gas in occupational settings^5^. Both NH$_{3}$ and H$_{2}$S are colorless, water- soluble, and flammable gases with distinct odors^6^. H$_{2}$S is highly toxic and corrosive, commonly found in sewers, wells, and various industrial processes^7^ and has a threshold limit value of 10 parts per million (ppm)^8^. OSHA has established safety limits for H$_{2}$S and NH$_{3}$, with a permissible exposure limit of 20 ppm and 25 ppm, respectively^9–12^. The prolonged exposure to these gases can pose various health problems to human beings^13–15^. Concentrations above 300 ppm are considered a serious threat to life and health^16^. Detecting and monitoring H$_{2}$S and NH$_{3}$ at low ppm levels is of the utmost importance to ensure the safety of individuals and prevent potentially fatal exposures. H$_{2}$S up to 0.1 ppm can be found in people with halitosis and diabetes, and it can be used as a biomarker to detect these diseases^17^. Similarly, infection due to kidney disease^18^ and Helicobacter pylori^19^ can result in an increase in NH$_{3}$ in exhaled breath, and it can be used as a biomarker to detect these diseases. Moreover, detecting these gases at room temperature in sub-ppm concentrations is highly desirable for applications such as detecting food spoilage and medical diagnostics through breath analysis.

Traditional methods like gas chromatography and mass spectrometry have been employed to measure low concentrations of H$_{2}$S and NH$_{3}$. However, these methods are bulky and costly and often involve complex sampling and analyzing processes^12^. Metal oxide (MOx) materials have gained widespread usage as active layers in the detection of NH$_{3}$^20,21^ and H$_{2}$S^22^. Although metal oxide (MOx)-based sensors are capable of sensing H$_{2}$S and NH$_{3}$ at subparts per million levels^23,24^, these sensors often exhibit drawbacks such as low selectivity, long response times, and high operating temperatures (>100 °C), which can hinder energy-efficient operation, portability, and real-time detection^12,25^. Taking into account these limitations, in recent years, conducting polymers (CPs) have emerged as a potential alternative to MOx materials for gas-sensing applications^26,27^.

Conducting polymers offer notable advantages, including remarkable mechanical properties, flexibility, ease of synthesis and modification through chemical or electrochemical processes, low cost, high sensitivities, and short response times with tunable optical and electronic characteristics^28,29^. One of the key advantages of CP-based gas sensors is their ability to operate effectively at room temperature^30^. The gas-sensing mechanism of conducting polymers relies on the doping and dedoping (oxidation/reduction) of the polymeric material. In particular, in electrochemical gas sensors, when analyte gas molecules come into contact with the working electrode, oxidation/reduction of the gas molecules occurs through a redox reaction, and the consequent flow of the charge carriers from the working electrode (anode/cathode) to the counter electrode through the conducting active layer causes an electric current^31^. In chemoresistive sensors, when analyte gas molecules come in contact with the active conducting layer, the resistivity/conductivity of the polymer changes depending on the conducting nature of the active layer material^32^. The p-type and n-type CPs, respectively, donate electrons to the analyte gas or receive electrons from the analyte gas, which increases the hole or electron conductivity, respectively. The free charge carriers then travel to the respective electrodes. The working principle of this kind of device is highly dependent on the interaction between the polymer and the analytes. The presence of $\pi$-conjugation in the backbone of the polymer is an essential factor for conductivity, and their interchain $\pi -\pi$ stacking enables the consecutive charge motion through the material.

Most studies on CP-based gas detection revolve around p-type conducting polymers such as polyaniline^33^, polypyrrole^34^, polythiophene^35^, and poly(3,4-ethylene dioxythiophene)^36^ and their composites due to the better stability of p-type polymers at ambient conditions than n-type polymers. However, p-type CPs exhibit limitations in terms of selectivity, stability, and sensitivity toward reducing gases like carbon monoxide (CO), ammonia (NH$_{3}$), and hydrogen sulfide (H$_{2}$S)^37^. As n-type conducting polymers lag behind in several electronic devices mainly due to their stability issue in ambient conditions, the usual n-type materials are generally limited to organic/inorganic nanoparticles, e.g., fullerene and its derivatives, TiO$_{2}$, and CeO$_{2}$^38–42^ and, hence, some CP-based gas sensors are made of a blend of p-type polymer and n-type nanoparticles^43^. One major drawback of using p-type CPs in sensing reducing gases is that the mechanism involves the dedoping of the p-type polymers, which in turn decreases the conductivity. Hence, the use of n-type materials has shown potential in detecting reducing gases as the conductivity increases when exposed to such gases^26^. This characteristic leads to enhanced selectivity and sensitivity toward reducing gases.

Despite the stability issues of most of the n-type polymers, ladder-type polybenzimidazole benzophenanthroline (BBL) shows remarkable stability and (opto)electronic properties and has shown potential to be used in organic electronics^44–47^. BBL exhibits high thermal stability up to $600^{\circ }C$ in air and $700^{\circ }C$ in an inert atmosphere^48^. Its mechanical properties include a high tensile strength of approximately 115 MPa^49^. Moreover, BBL possesses a crystalline structure with a linear $\pi$-conjugated backbone unit and a redox site^50^. In particular, BBL demonstrates very high electron mobility of around $0.1 cm^{2}sV^{-1}$^51^, which further contributes to its suitability as an ideal n-type polymer for sensing reducing gases.

The sensing mechanism in CP-based chemo-resistive gas sensors includes the interaction of gas with the polymers, followed by the transfer of charges from the analyte gas to the polymer and, ultimately, the transport of charge carriers through the crystalline network of the polymer film. Often, the smooth diffusion of the gases through the active layer and the consequent adsorption of the gas molecules on polymers resulting from the strong interaction between the analytes and polymers are crucial and the first criteria for selectively and rapidly detecting the gas. Typically, the adsorption exhibited by these sensors is in the range of physisorption and depends on the number of active sites in the polymer matrix as well as the morphology of the polymer. After the analyte gases are adsorbed on the surface of the polymer, they can undergo charge transfer based on the reducing/oxidizing nature of the analyte gas. The crystallinity of the material plays an important role as the interchain $\pi -\pi$ stacking of the polymers enables the consecutive motion of the charge carriers through the active layer. The morphological characteristic can impact both the sensitivity and response time of the sensor^52^. H$_{2}$S and NH$_{3}$ are both reducing gases that can negatively dope the film and increase the electrical conductivity of n-type polymers by providing more electrons as majority charge carriers to the polymer film^26^. However, sensors made of n-type conducting polymers are missing to a great extent, and the best of our knowledge, there is no reported study on gas sensing using n-type CP. The potential of BBL has recently been explored as an organic electrochemical transistor-based sensor for glucose detection^53^. However, a thorough understanding of the chemo-resistive properties of BBL thin films and their interaction with the analytes is lacking; therefore, its gas sensing-related applications have not yet been explored.

The computational tools, including both density functional theory (DFT) and molecular dynamics (MD) simulations, can provide a detailed understanding of the interaction of gas molecules with the sensing material. The DFT approach has been used to study the binding energies of various gas molecules on the inorganic and organic materials and their resulting changes in geometric and electronic structures to understand the sensing performance^54,55^. The MD simulations were employed to explore the interaction and diffusion of various gases such as H$_{2}$, CH$_{4}$, NH$_{3}$, SO$_{2}$, SO$_{3}$ etc. on materials such as metal oxide (TiO$_{2}$ and TiO$_{2}$:MoO$_{3}$) surfaces, graphene, conducting polymer such as polyaniline and its derivatives^56–59^.

In the present study, we provide insights into the potential of the BBL for detection of H$_{2}$S and NH$_{3}$ by calculating the strength of interaction energy and the magnitude of charge transfer (CT) using the DFT approach. The higher binding affinity and higher CT of NH$_{3}$ compared to H$_{2}$S indicates that the BBL is more selective and sensitive towards NH$_{3}$ than H$_{2}$S. In addition, using MD simulation, we reveal that the adsorption of both gases takes place inside the void volume of the BBL and does not have a significant impact on morphological changes associated with the gas adsorption within the BBL matrix by analyzing the X-ray diffraction (XRD), radial distribution function (g(r)) and the bulk interaction energy between the H$_{2}$S or NH$_{3}$ molecules and BBL chains. Our calculations provide us with valuable insights into the microscopic details of the interaction between H$_{2}$S/NH$_{3}$ and the BBL and can be a guide in fabricating an n-type CP-based gas sensor for reducing gases.

## Results

### Morphology analysis of BBL film before and after gas exposure

**Fig. 1:**
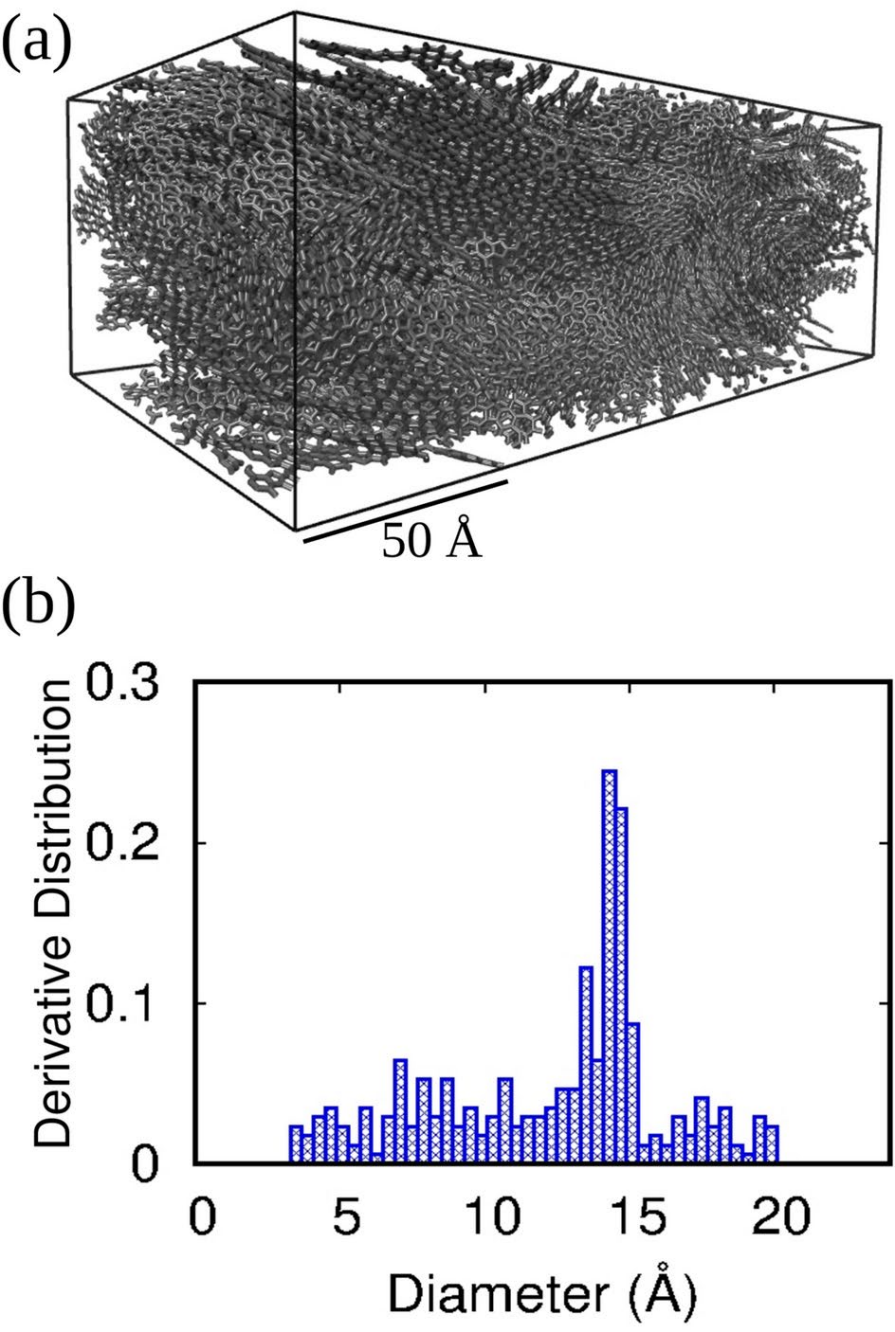
(**a**) Representative snapshot, and (**b**) pore size distribution (PSD) of unloaded BBL film.

First, we analyzed the morphology of the unloaded BBL film prepared through molecular dynamics simulations and evaluated its potential to be used as an active material for gas sensing. The MD-prepared unloaded BBL film is shown in Fig. 1a. For better gas sensing performance, the material should be porous so that the analyte gases can easily diffuse through it. Besides, it should also be a promising adsorbent with a high effective surface area, free volume, and higher binding energy. We have analyzed the pore size distribution (PSD) of unloaded BBL film as shown in Fig. 1b. The pore size distribution reveals that the BBL film is a nanoporous material with porosity ranging from 5 to 20 Å. Moreover, as the size distributions of the pores are less than 2 nm, this can be further categorized as microporous material^60^. The calculated available volume and surface area of the unloaded BBL film are 6.68 $\times 10^{-8}$ m$^{3}$/g and 332.339 m$^{2}$/g, respectively. After exposure to the analyte gases, the calculated free volumes for the BBL-H$_{2}$S and BBL-NH$_{3}$ systems are 3.08 $\times 10^{-8}$ m$^{3}$/g and 6.31 $\times 10^{-8}$ m$^{3}$/g, respectively, see Supplementary Table S2. Previous studies on polyaniline/SrGe$_{4}$O$_{9}$ nanocomposites (PSN) used for ammonia sensing reported a surface area of 21.3 m$^{2}$/g^61^. Additionally, the BET surface area of graphene oxide (GO) sheets used for ammonia sensing is 37.24 m$^{2}$/g^62^. Compared to previously reported materials for ammonia sensing, these results signify that the pristine BBL film exhibits a nanoporous structure with high free volume and surface area, suitable for the adsorption and faster diffusion of gas molecules. As a result, BBL film, as an active material, can show better performance in gas-sensing applications.

**Fig. 2:**
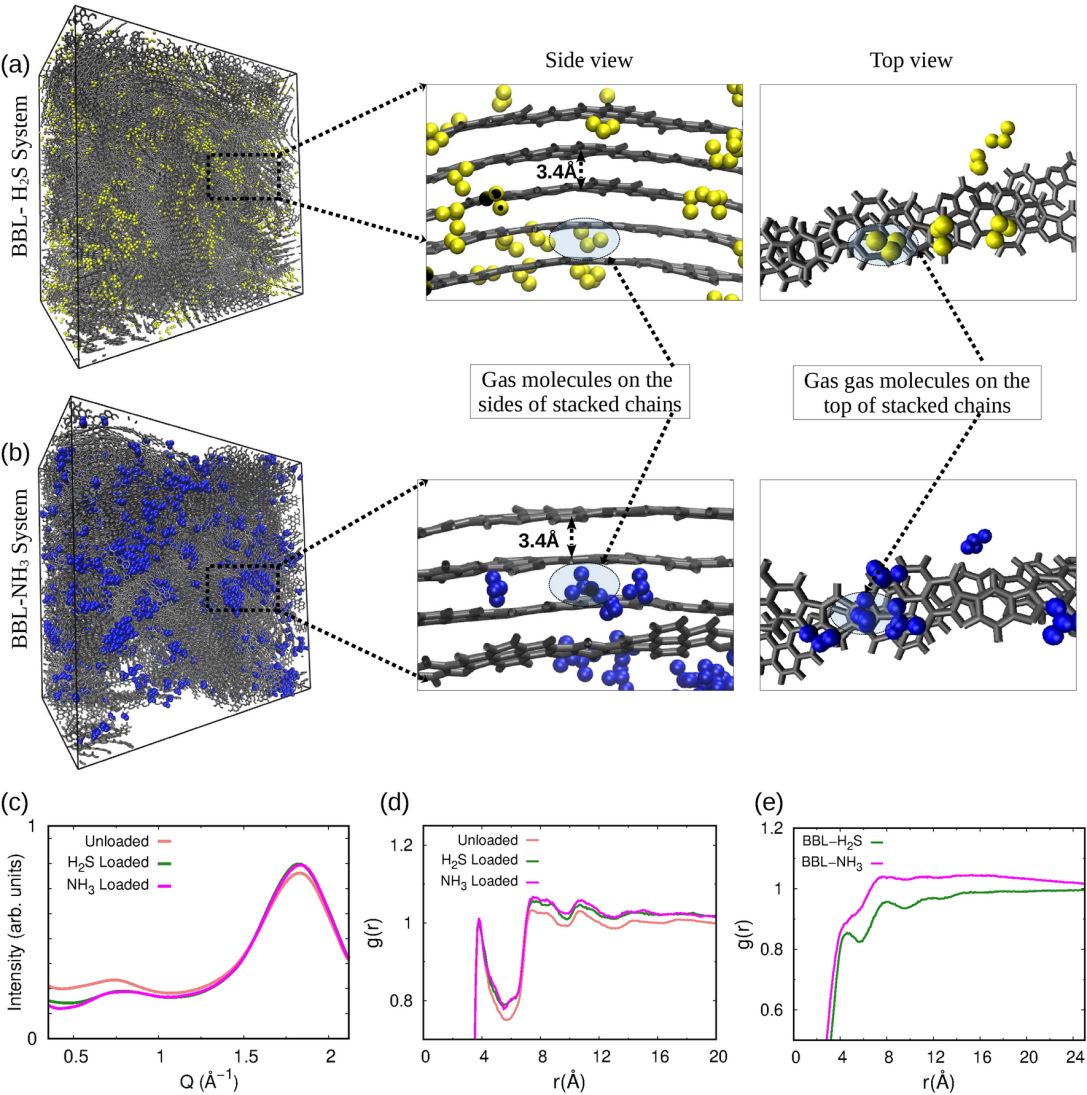
The snapshots of MD-equilibrated (**a**) BBL-H$_{2}$S and (**b**) BBL-NH$_{3}$ system with the corresponding zoomed view of $\pi$-$\pi$ stacked BBL chains in the presence of the respective gases, (**c**) XRD plot for unloaded BBL, H$_{2}$S loaded and NH$_{3}$ loaded systems, (**d**) radial distribution function g(r) of BBL chains of unloaded, H$_{2}$S loaded and NH$_{3}$ loaded systems, and (**d**) *g*(*r*) of H$_{2}$S and NH$_{3}$ gas molecules around the polymers.

Next, we examined the microscopic morphological changes of the BBL film after separate exposure to both gases, H$_{2}$S and NH$_{3}$. Representative snapshots of the MD simulated BBL- H$_{2}$S and BBL- NH$_{3}$ systems are shown in Fig. 2a, b. Through visual inspection, we note that the H$_{2}$S and NH$_{3}$ are occupied primarily in the spaces between and around polymer crystallites, i.e., regions with partial ordering, rather than in the spaces between the $\pi$-$\pi$ and lamellar—stacked chains and the overall structure of the polymer film remained almost unaltered upon gas adsorption without any apparent swelling (see the Supplementary Information for more details) or significant morphological changes in the film, compared to the unloaded film, discussed as follows. We identified the presence of 3–4 aggregates of $\pi$-$\pi$ stacked chains, referred to as crystallites, and their network throughout the film, by visual inspection as shown in Fig. 2a, b and Supplementary Fig. S1b, which would facilitate the transport of charge carriers upon reduction of the BBL polymers during the device operation. In order to quantify the regions with partial ordering in the film, we calculated the XRD and radial distribution function, *g*(*r*) of the BBL film obtained from the MD simulations before and after gas adsorption, as shown in Fig. 2c, d. To validate the MD simulations, we compared the simulated XRD with the experimental XRD of the unloaded BBL and found that the simulated XRD is in line with the experimental XRD, see Supplementary Fig. S1a.

All the XRD patterns show a peak at Q = 0.75 Å$^{-1}$ corresponds to a distance of d = 2$\pi$/Q = 8.37 Å, accounts for the lamellar stacking and a sharp peak at approximately Q = 1.8 Å$^{-1}$ corresponds to a distance of d = 3.39 Å, accounts for the $\pi$-$\pi$ stacking, see Fig. 2c and Fig. S1a. The d-spacing of lamellar and $\pi$-$\pi$ stacks remain almost the same for the loaded and unloaded films, indicating that the BBL polymer maintains its crystalline structure even after interacting with both gases, see Fig. 2c. We note, there is a slight increase in intensity in the $\pi$-$\pi$ stacking peak in the XRD of the loaded films, indicating that the number of $\pi$-$\pi$ stacks increases slightly after gas adsorption compared to unloaded BBL, and the same trend is observed in *g*(*r*) of BBL polymers, see Fig. 2d. The g(r) of both loaded and unloaded BBL film show 4–5 peaks in the multiple of $\pi -\pi$ stacking distance. It is worth mentioning that depending on the orientation of the two chains in the $\pi -\pi$ stack, the $\pi$-conjugated polymers can show eclipsed, i.e., cofacial $\pi -\pi$ stacking where two chains are exactly on top of each other with a negligible slip, and they can also show slipped $\pi -\pi$ stacking where one chain is not exactly on top of another chain possessing longitudinal and/or transverse shifts^63,64^. The cofacial eclipsed stacking forms when two polymer chains are aligned in the opposite direction (AB), and slipped staggered stacking forms when two polymer chains are aligned in the same direction (AA) due to the repulsion between like charges between the atoms in the polymer chains^63,64^, as shown in Fig. S2a. In the MD-prepared film, we observed the formation of both types of $\pi$-stackings, and the DFT calculations show that both the structures have almost the same energy, discussed in detail in Supplementary Information, see Section S1.2.

We further quantified the crystallinity with respect to the $\pi$–$\pi$ stacked BBL chains by calculating the crystallite size from the XRD plot for both the loaded and unloaded film, which confirms, although the crystallite size slightly increases, but the average number of polymers in a crystallite almost remains the same as shown in Supplementary Table S1, which is also visible from the number of spikes in *g*(*r*) of BBL polymers in Fig. 2d. The slight decrease in intensity in the lamellar stacking peak is because of the structural rearrangement due to the uptake of the gas molecules, see Fig. 2c. Furthermore, we note, upon gas uptake, there is an increase in the peak height of the $\pi$–$\pi$ stacking peak in the XRD and an increase in the crystallite size. It is noteworthy to mention here that the number of $\pi$–$\pi$-stacked chains with short stacking distance is one of the predominant factors in $\pi$-conjugated conducting polymers for the mobility of charge carriers^65,66^ and the increase in number of $\pi$–$\pi$-stacks will help in increasing sensitivity of the polymer upon exposure to gaseous molecules because of the easy transport of free charge carriers generated by the redox reaction between the polymer and the analyte. The experimentally measured FTIR results indicate the film is not chemically altered after exposure to the ammonia gas, see Fig. S4, indicating the effectiveness of repeatability of the material in operation. More details on the FTIR characterization are provided in the Supplementary Information, see Section S2.

Next, we analyzed the radial distribution function, *g*(*r*) of the gas molecules around the polymers to understand interactions between polymers and gases, shown in Fig. 2e. It provides valuable microscopic insights, indicating the likelihood of finding gas molecules at a distance from the polymer chains. The distinct peaks in both the *g*(*r*) at $\sim$ 4.5 Å, $\sim$ 8 Å, and $\sim$ 11.5 Å reveal that both gases predominantly interact with the polymer and show multilayer adsorption, but at the same time, leaving the crystalline structure of the BBL thin film unaffected, as seen in the XRD of the film. Apparently, more intense peaks in *g*(*r*) of the NH$_{3}$ gas around the BBL chain reveal that a more significant number of adsorbed NH$_{3}$ molecules are more likely to be located near polymer chains than H$_{2}$S molecules indicating a stronger interaction between NH$_{3}$ and BBL than H$_{2}$S and BBL. To delve deeper into the interaction energy, we have calculated the interaction energies of gas molecules with the polymer using MD simulations and compared them with adsorption energies calculated using DFT. We have also calculated the preferred gas adsorption sites, the magnitude of charge transfer, the density of states, and the UV- vis absorption spectra using DFT, as discussed in the following section.

### Interaction energy and preferential adsorption sites

**Fig. 3:**
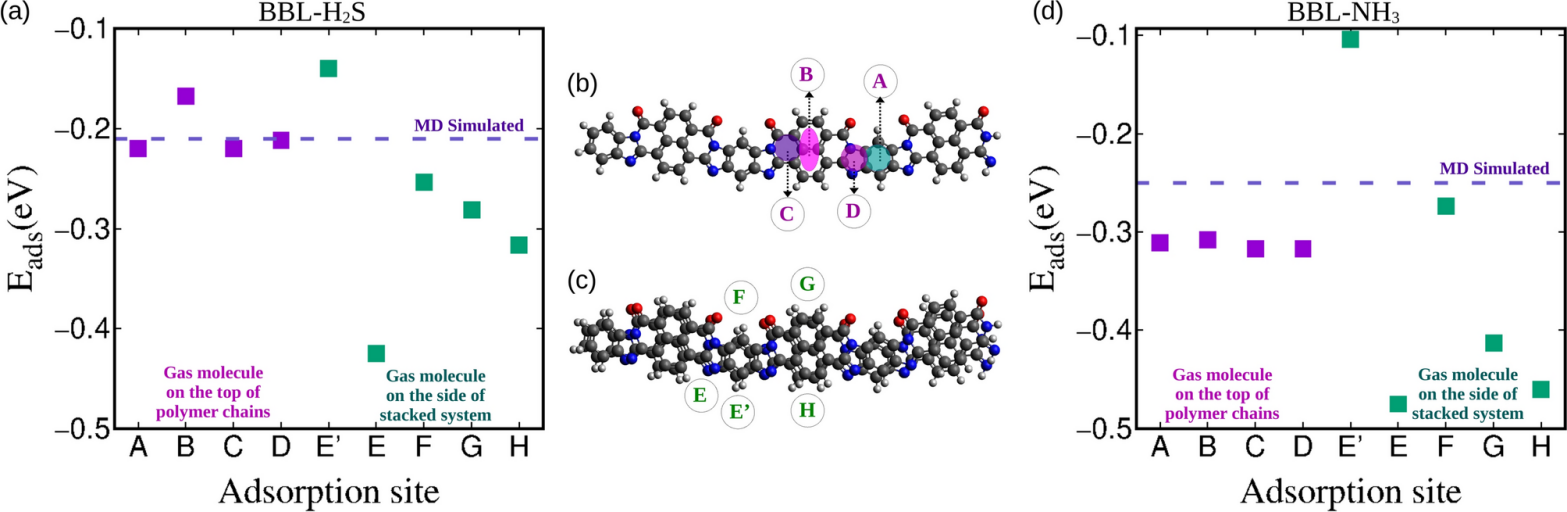
(**a**) DFT calculated adsorption energies ($E_{ads}$) of different adsorption sites for BBL-H$_{2}$S system and comparison with MD calculated $E_{ads}$, (**b**) adsorption sites considered on top of different rings present in a BBL polymer chain, (**c**) different adsorption sites considered on the sides of a stack consist of two BBL polymer chains and (d) DFT calculated adsorption energies ($E_{ads}$) of different adsorption sites for BBL-NH$_{3}$ system and comparison with MD calculated $E_{ads}$

We observe from the MD-equilibrated BBL-H$_{2}$S and BBL-NH$_{3}$ systems that the gas molecules are adsorbed both on the top and sides of the $\pi -\pi$ stacked BBL chains, see Fig. 2a, b. We have calculated the intermolecular interaction energy (E$_{ads}$) between BBL and H$_{2}$S and BBL and NH$_{3}$ from the MD simulations, which include both van der Waals (E$_{vdW}$) and Coulombic interactions (E$_{Coul}$) and is the average energy considering the gas adsorption in different adsorption sites, shown in Fig. 3. The van der Waals energy (E$_{vdW}$), Coulombic (E$_{Coul}$) interaction energies between BBL and H$_{2}$S/NH$_{3}$ are shown in Table 1. The E$_{ads}$, E$_{vdW}$, E$_{Coul}$ between BBL and gas molecules calculated using MD simulations show higher strength of interactions for BBL towards NH$_{3}$ than H$_{2}$S.

**Table 1:** The van der Waals energy (E$_{vdW}$), Coulombic (E$_{Coul}$) interaction energies between BBL and H$_{2}$S/NH$_{3}$ per gas molecules (H$_{2}$S and NH$_{3}$) and the corrresponding Diffusion Coefficient (*D*) on BBL polymer, obtained from MD simulations

Gas	E$_{vdW}$ (eV)	E$_{Coul}$(eV)	*D* (cm$^{2}$/s)
H$_{2}$S	– 0.21	– 2.7 $\times$ 10$^{-3}$	2.8 $\times$ 10$^{-7}$
NH$_{3}$	– 0.25	– 5.07 $\times$ 10$^{-3}$	0.96 $\times$ 10$^{-7}$

To further assess the strength of interaction between the gas molecules and the BBL polymer chain, we calculated the adsorption energy (E$_{ads}$), which is defined by the difference between the total energy of the adsorbent-adsorbate complex and the energy of isolated adsorbate and adsorbent molecules, of both the reducing gas molecules, H$_{2}$S and NH$_{3}$ with a BBL polymer chain using the DFT level of quantum mechanical calculation. We have calculated E$_{ads}$ of four different sites by placing the gas molecules at the top of a BBL chain, viz., on benzene (A), naphthalene (B), pyridine (C), and imidazole (D) rings, and of another four adsorption sites by placing the gas molecules at the sides of two $\pi -\pi$ stacked BBL chains, viz., close to oxygen-lean (E$^\prime$) and oxygen-rich (F) side of benzene and oxygen-rich (G) and oxygen-lean (H) side of naphthalene, and oxygen-lean side of imidazole ring (E), as shown in Fig. 3b, c. The DFT calculated E$_{ads}$ of all the sites and the MD-simulated E$_{ads}$ which is the average adsorption energy, for H$_{2}$S and NH$_{3}$ are shown in Fig. 3a and d, respectively. The comparison of MD-simulated energy for both BBL-H$_{2}$S and BBL-NH$_{3}$ systems with the DFT calculated adsorption energies considering the energies of different adsorption sites, along with the zoomed-in snapshots of MD systems shown in Fig. 2a, b, indicate that the adsorption of gas molecules takes place at different top and side adsorption sites of BBL rather than only at the sites having the highest $\mid E_{ads} \mid$, in the case of bulk adsorption.

**Fig. 4:**
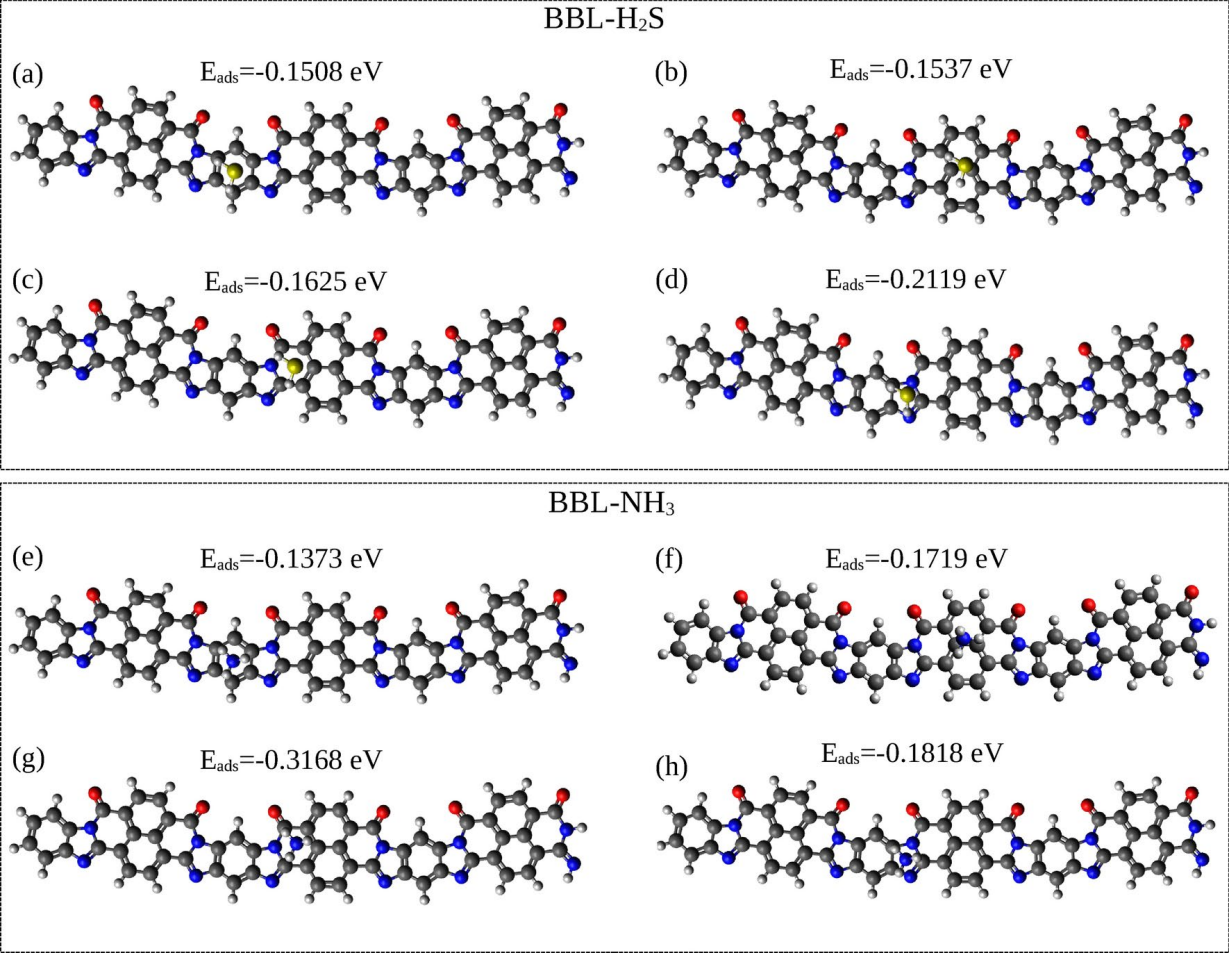
[Upper Panel] (**a**–**d**) The corresponding adsorption energies (E$_{ads}$) of one H$_{2}$S molecule when adsorbed on four different adsorption sites on top of a BBL trimer, namely, benzene, naphthalene, pyridine, imidazole, respectively, along with the geometries. [Lower Panel] (**e**–**h**) The corresponding E$_{ads}$ of one NH$_{3}$ molecule when adsorbed on four different adsorption sites on a BBL trimer, namely, benzene, naphthalene, pyridine, imidazole, respectively, along with the respective geometries (top view).

The adsorption energies along with the corresponding geometries (top view) of one H$_{2}$S and one NH$_{3}$ molecule placed separately at different sites at the top of the single BBL chain are shown in Fig. 4a–d and e–h, respectively. (The side view of the geometries for all the adsorption sites identified on top of the BBL chain is shown in Fig. S5). The calculations show that one H$_{2}$S molecule placed on an imidazole ring has the highest $|E_{ads} |$, and an NH$_{3}$ placed on a pyridine ring has the highest $|E_{ads} |$. Furthermore, upon geometry optimization of the BBL-H$_{2}$S complex, when H$_{2}$S is placed on top of BBL chain at different adsorption sites, the H$_{2}$S molecule finally gets adsorbed on an imidazole ring except when placed on a naphthalene ring with a lower $|E_{ads} |$ than the imidazole ring, as shown in Table S3. Upon optimization of the geometry by placing the NH$_{3}$ molecule at different sites of BBL, it always gets adsorbed on the pyridine ring, see Supplementary Table S4. Hence, we note that the imidazole and pyridine rings are the respective preferential adsorption sites for H$_{2}$S and NH$_{3}$ when they are adsorbed on top of a BBL chain. Note, in the film made of BBL, the chains show $\pi -\pi$ stackings. We calculated the adsorption energies placing the gas molecules at the top of two $\pi -\pi$ stacked chains and found a negligible difference in $E_{ads}$ compared to the single BBL chain, see Table S5 in Supplementary Information.

**Fig.5:**
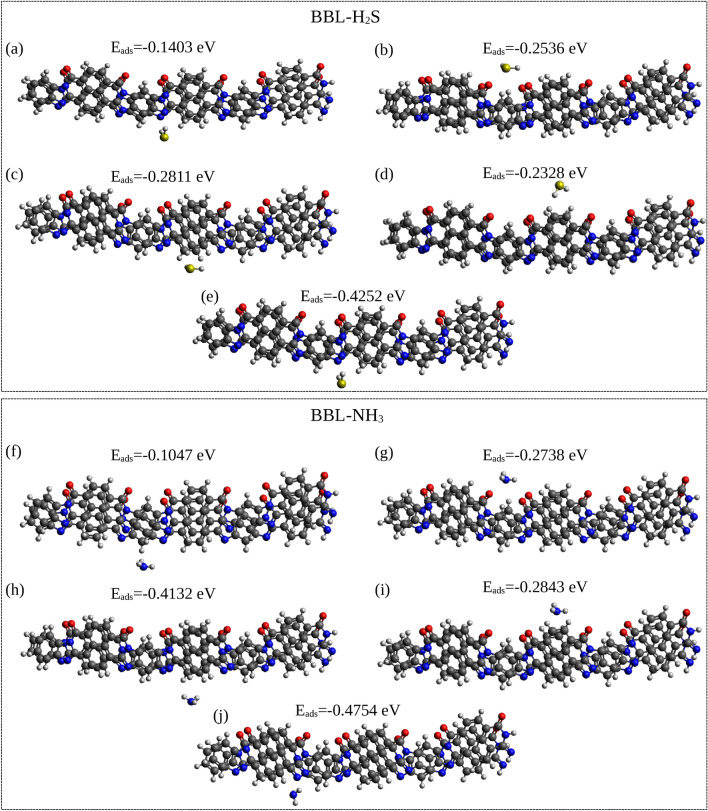
[Upper Panel] (**a**–**e**) The corresponding adsorption energies (E$_{ads}$) of one H$_{2}$S molecule placed on four different adsorption sites on the sides of stacked BBL chains, namely, close to oxygen-lean (E$^\prime$) and oxygen-rich (F) side of benzene and oxygen-rich (G) and oxygen-lean (H) side of naphthalene, and oxygen-lean side of imidazole ring (E), respectively, along with the geometries. [Lower Panel] (**f**–**j**) The corresponding E$_{ads}$ of one NH$_{3}$ molecule placed on four different adsorption sites on the sides of stacked BBL chains, namely, close to oxygen-lean (E$^\prime$) and oxygen-rich (F) side of benzene and oxygen-rich (G) and oxygen-lean (H) side of naphthalene, and oxygen-lean side of imidazole ring (E), respectively, along with the geometries (top view).

Next, we explore the possible adsorption sites on the sides of $\pi -\pi$ stacked BBL chains, as shown in Fig. 5. (The side view of the geometries for all the adsorption sites on the sides of $\pi -\pi$ stacked BBL chains is shown in Fig. S7.)

For both BBL-H$_{2}$S and BBL-NH$_{3}$ systems, the preferred adsorption site was found to be the site near an imidazole ring on the oxygen-lean side, viz., site *E*, see Figs. 3a, d and 5e, j. The adsorption energies $E_{ads}$ and the corresponding intermolecular equilibrium distances * d* between the gas molecules and the BBL polymer chain of the preferential adsorption sites for the gas molecules at the top and side sites of the BBL chains found from the DFT calculations are summarized in Table 2. The calculated adsorption energy of the preferential site at the top of a $\pi -\pi$ stack for H$_{2}$S and NH$_{3}$ are – 0.22 eV and – 0.33 eV, respectively, and are adsorbed at a distance, (*d*$_{N/S}$) of 3.74 Å and 3.23 Å, respectively, (Discussed in detail in the Supplementary Information). The calculated adsorption energy of the preferential site at the sides of a $\pi -\pi$ stack for H$_{2}$S and NH$_{3}$ are – 0.42 eV and – 0.47 eV, respectively, and are adsorbed at a distance, (*d*$_{N/S}$) of 3.48 Å and 3.13 Å, respectively. We note, BBL has a higher strength of interaction, i.e., higher $|E_{ads} |$, smaller interaction distance, and higher magnitude of charge transfer for NH$_{3}$ compared to the H$_{2}$S, which indicates that NH$_{3}$ gas has a higher affinity for the BBL polymer than the H$_{2}$S gas.

We have also calculated the interaction of the BBL chain with other common gases present in the air, such as O$_{2}$, N$_{2}$, CO$_{2}$, and H$_{2}$O, see Supplementary Fig. S9 and Fig. S10. From $E_{ads}$ we note, both H$_{2}$S and NH$_{3}$ are more selective towards BBL compared to N$_{2}$ and O$_{2}$, which are present in high concentrations in air. Moreover, NH$_{3}$ has the highest strength of interaction energy compared to all other gases commonly present in the air, as shown in Fig. S10.

**Table 2:** Geometries of H$_{2}$S molecule placed on the preferential adsorption sites at the top and side of stacked BBL polymer chains along with the corresponding adsorption energies (E$_{ads}$), equilibrium distance (d$_{N/S}$), the magnitude of charge transfer (CT), and the corresponding band gap (E$_{g}$). d$_{N/S}$ (Å) represents the equilibrium distances of the Nitrogen/Sulfur atoms of NH$_{3}$/H$_{2}$S from the centre of mass of the adsorption site.

Analyte gas	Optimized geometry	E$_\textrm{ads}$ (eV)	dN/S (Å)	CT	Eg (eV)
H$_2$S	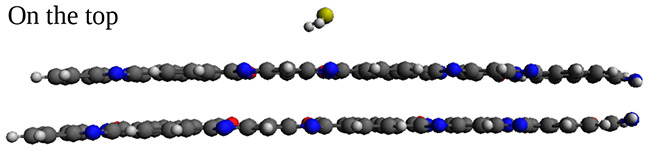	– 0.22	3.74	0.00097	5.70
	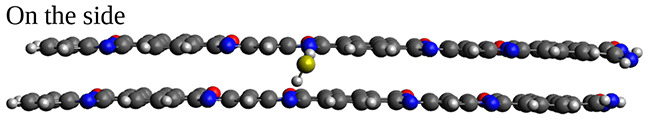	– 0.42	3.48	0.00058	5.65
NH$_3$	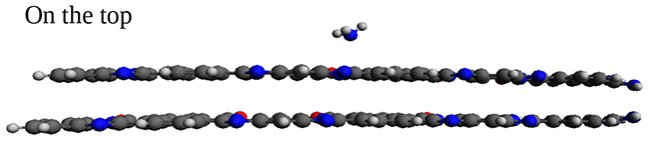	– 0.33	3.23	0.01460	5.65
	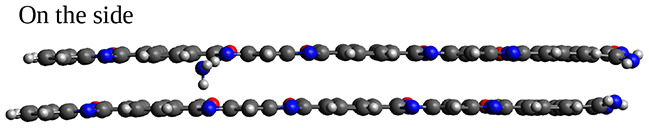	– 0.47	3.13	0.03700	5.61

We note, from the visual inspection of adsorption sites from the snapshots of the MD-equilibrated system and the interaction energy calculations, as shown in Figs. 2a, b and 3, respectively, that in the bulk systems, the adsorptions take place at both top and sides of the BBL chain and the change in (opto)electronic properties should have contributions from both the top-site and side-site adsorption. Therefore, to further analyze the systems in terms of their electronic and optical properties in presence of the H$_{2}$S and NH$_{3}$ gases, we considered both the top and side adsorption sites.

Next, we assess the intermolecular electronic interactions of BBL-H$_{2}$S and BBL-NH$_{3}$ complexes, where the H$_{2}$S and NH$_{3}$ gases are adsorbed at the preferential adsorption site on top and sides of BBL, by calculating the charge transfer magnitude between the gas molecules and the BBL polymer chain, which is investigated using the Natural Bond Orbital (NBO) analysis^67,68^. The NBO analysis, which provides the electronic density distribution of atoms and bonds, is considered less dependent on the basis set than Mulliken populations and is an important tool to study intermolecular electronic interactions^68^. The natural bond orbital charge distributions of an isolated BBL chain, BBL-H$_{2}$S complex, and BBL-NH$_{3}$ complex are shown in Fig. S8. The calculations show, for the BBL-H$_{2}$S complex, the BBL polymer acquires a negative charge of 0.00097 and 0.00058, and the gas molecule acquires a positive charge of the same magnitude when placed on top and side preferential sites of BBL, respectively, tabulated in Table 2. On the other hand, for the BBL-NH$_{3}$ complex, the BBL polymer acquires a negative charge of magnitude 0.01460 and 0.03700, and the gas molecule acquires a positive charge of the same magnitude when placed on top and side preferential sites of BBL, respectively. The higher magnitude of charge transfer (CT) for the BBL-NH$_{3}$ system compared to the BBL-H$_{2}$S system indicates that the BBL has a higher sensitivity for NH$_{3}$ than for H$_{2}$S. Moreover, the BBL-NH$_{3}$ system shows significantly better charge transfer values than previously reported 0.023 for ZnO^69^, which is a metal oxide-based gas sensor used for NH$_{3}$ detection. Also, it is noteworthy that NH$_{3}$ exhibits a superior charge transfer value with BBL compared to other common gases in the air, as shown in Supplementary Fig. S10. We compared the H$_{2}$S/NH$_{3}$ adsorption on top of an isolated chain with that of a $\pi -\pi$ stack made of two BBL chains, in terms of adsorption energy, magnitude of charge transfer, band gap, and the evolution of density of states, as shown in Fig. S6 and Table S5 (see Section S3.1.1 in the Supplementary Information). We found that for the top-site adsorption, a single chain can predict the system’s behavior similar to that of the $\pi -\pi$ stacked chains. However, to study the side-site adsorptions, the consideration of a $\pi -\pi$ stack is required as the adsorption sites are formed on the sides of two $\pi -\pi$ stacked chains.

**Fig. 6:**
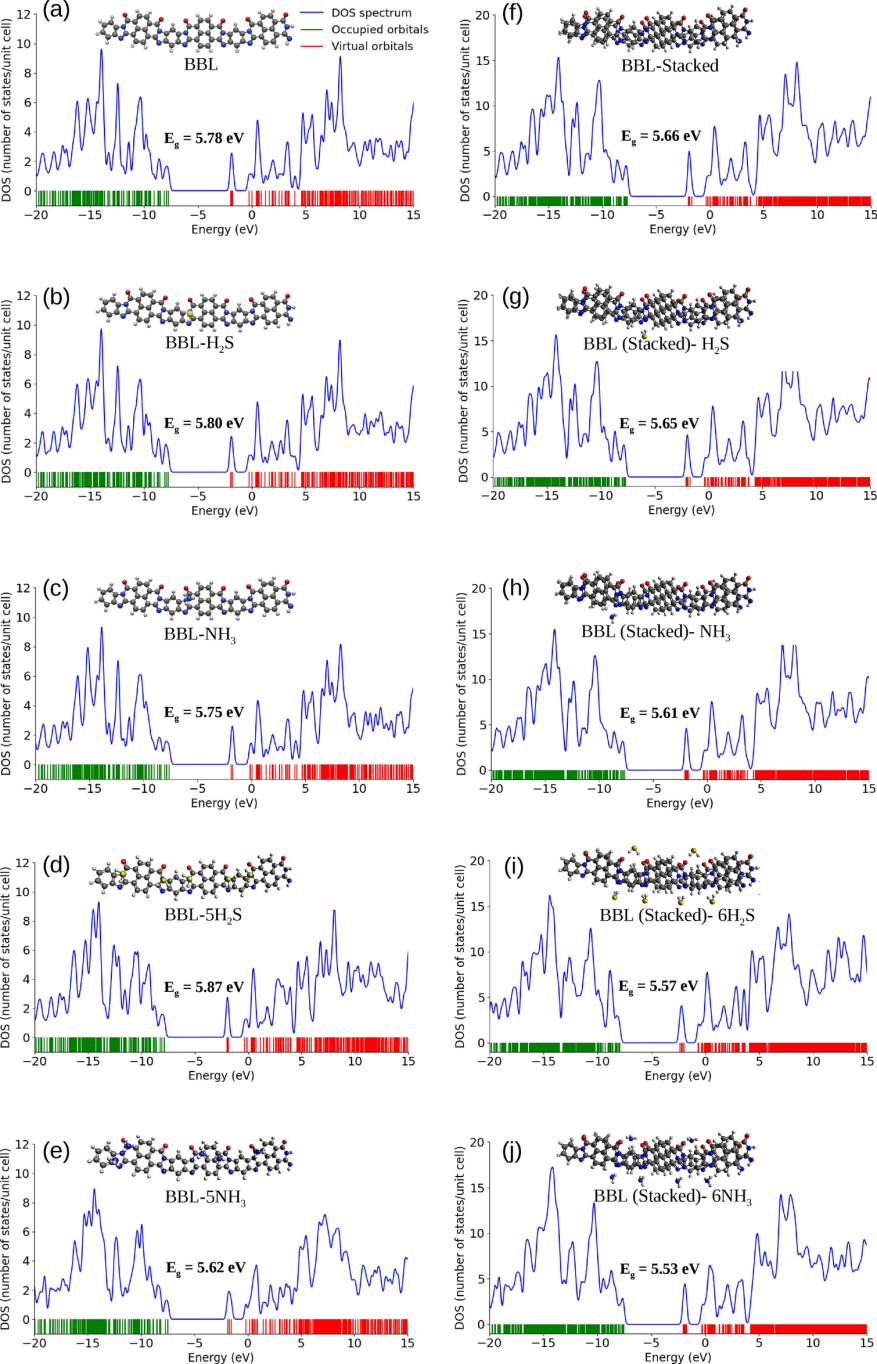
[Left panel] Density of States (DOS) along with the optimized geometry of (**a**) isolated BBL chain, (**b**) BBL-H$_{2}$S complex, (**c**) BBL-NH$_{3}$ complex, (**d**) BBL-5H$_{2}$S complex, and (**e**) BBL-5NH$_{3}$ complex. [Right panel] Density of States (DOS) along with the optimized geometry of (**f**) stacked BBL chains, (**g**) BBL (stacked)-H$_{2}$S complex, (**h**) BBL (stacked)-NH$_{3}$ complex, (**i**) BBL (stacked)-6H$_{2}$S complex, and (**j**) BBL (stacked)-6NH$_{3}$ complex.

To understand the effect of the gases, H$_{2}$S and NH$_{3}$, on the electronic structure of the BBL polymer in the ground state, we plot the density of states (DOS) of BBL, BBL with H$_{2}$S molecule, and BBL with NH$_{3}$ molecule as shown in Fig. 6a–c and f–h. The DOS of isolated H$_{2}$S and NH$_{3}$ molecules are shown in Supplementary Fig. S11. The occupied energy levels in the valence band are shown in green, and the virtual energy levels in the conduction band are shown in red. The energy gap (E$_g$) between the two orbitals is marked for all three systems within the figure. The DFT-calculated band gap, defined as the energy difference between the highest occupied molecular orbital and the lowest unoccupied molecular orbital, does not represent the actual value of the bandgap of the polymer^70^. However, the energy levels in the valence/conduction band obtained from the DFT calculations can qualitatively predict the system’s nature^71,72^. We note, there are minute changes in the ground state electronic structure of the BBL chain in the presence of the two gas molecules, which in turn results in a change in E$_g$. For the case of NH$_{3}$ adsorbed at the preferential site on top of BBL, the E$_g$ slightly decreases, whereas for the case of H$_{2}$S molecule adsorbed at the preferential site on top of BBL, the E$_g$ increases slightly, also shown in Supplementary Fig. S12. This change is more significant for the case of multiple adsorbed gas molecules per chain, see Fig. 6d, e. For the gas molecules adsorbed at the preferential site on the side of a $\pi -\pi$ stack, for both H$_2$S and NH$_3$, E$_g$ slightly decreases, but the change is clearly visible for the case of multiple gases and further, the change is more for BBL-NH$_3$, see Fig. 6f–j. To see the effect of multiple gas molecules adsorbed on top and sides of BBL, we placed one H$_{2}$S/NH$_{3}$ molecule on each preferential site (total 5 for top-sites and total 6 for side-sites), neglecting the last imidazole/pyridine ring to minimize the finite size effect of the chain length, see Fig. 6d, e and i, j.

**Fig. 7:**
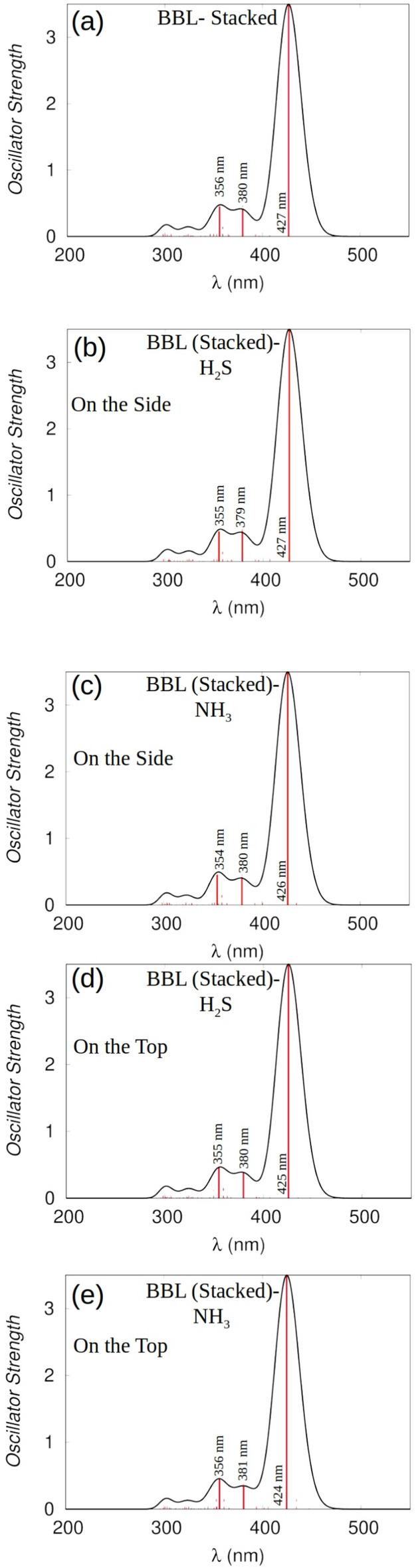
UV-vis absorption spectra of (**a**) stacked BBL chains, (**b**) H$_{2}$S, and (**c**) NH$_{3}$ gases on the side of the BBL (stacked) chains, (**d**) H$_{2}$S, and (**e**) NH$_{3}$ gases on the top of the BBL (stacked) chains.

The DOS calculation of the individual gas molecules shows that the HOMO energy level of NH$_3$ is higher than that of H$_2$S, HOMO$_{NH_3}$ (– 9.15 eV) >HOMO$_{H_2S}$ (– 9.32 eV), which indicates the electron donating strength of NH$_{3}$ is higher than H$_2$S, see Supplementary Fig. S11. This is in line with the CT calculation, which shows that the magnitude of charge transfer is more between BBL and NH$_{3}$ compared to BBL and H$_{2}$S, which could also be understood from the fundamental nature of NH$_{3}$ to be more basic than H$_{2}$S. On the contrary, the higher-lying LUMO of NH$_{3}$ (4.64 eV) compared to the LUMO energy level of H$_{2}$S (2.17 eV) indicates NH$_{3}$ would be a poorer electron acceptor than H$_{2}$S, see Supplementary Fig. S11. Indubitably, the LUMO energy levels of both the gas molecules lie very high compared to that of BBL. This clearly indicates that during the interaction of these gas molecules with BBL, BBL would act as an electron acceptor, and H$_{2}$S/NH$_{3}$ would act as an electron donor. We also note that the E$_g$ of H$_{2}$S and NH$_{3}$ are very large, and the interaction with both the gas molecules doesn’t significantly alter the BBL polymer’s electronic configuration. For more information, please see the Supplementary Information (Supplementary Fig. S12 and S13).

Next, we analyze the UV-vis absorption spectra of two $\pi -\pi$ stacked BBL chains, shown in Fig. 7(a) and UV-vis absorption spectra of BBL-H$_{2}$S, and BBL-NH$_{3}$ complexes, where the H$_{2}$S and NH$_{3}$ gases are adsorbed on the preferential adsorption site on side and top of BBL, as shown in Fig. 7b, c and d, e, respectively. The absorption spectra of a $\pi -\pi$ stack after gas adsorption for all the cases remain almost unaltered compared to that of the unloaded BBL. However, the spectra of the isolated BBL chain are different than the $\pi -\pi$ stacked chains, and the evolution of spectra after gas adsorption also shows a different trend where we observed diminishing of the first peak at 238 nm, upon interacting with the gas molecules, H$_{2}$S and NH$_{3}$, see Fig. S15, discussed in the Supplementary Information in detail. The presence of an isolated chain in the film represents the disorder, and if the degree of disorder in the film increases, the change in spectra that we observed when gas molecules are adsorbed on an isolated chain should contribute more to the overall spectra of the film.

The adsorption energy and the magnitude of charge transfer between H$_{2}$S/NH$_{3}$ and BBL show the potential of BBL to be used as an active material of gas sensors to detect H$_{2}$S and NH$_{3}$ and the calculations indicate better selectivity and sensitivity for NH$_{3}$ than H$_{2}$S. However, the evolution of electronic structures and optical properties of BBL upon interacting with H$_{2}$S and NH$_{3}$ gas molecules show a minute change in energy band gap and almost no change in the absorption spectra compared to that of the pristine BBL. But, note, during this study, we have considered the H$_{2}$S/NH$_{3}$ gas molecules have not undergone the redox reactions that cause the generations of free electrons and consequent acceptance of the electrons by the BBL. Further study, involving the oxidation of H$_{2}$S/NH$_{3}$ gas molecules and consecutive reduction of BBL^73^ and the evolution of (opto)electronic properties due to the formation of (bi)polaron species^71^ in reduced BBL needs to be carried out to understand the performance of BBL as electronic/optical gas sensor.

### Gas diffusion study

**Fig. 8:**
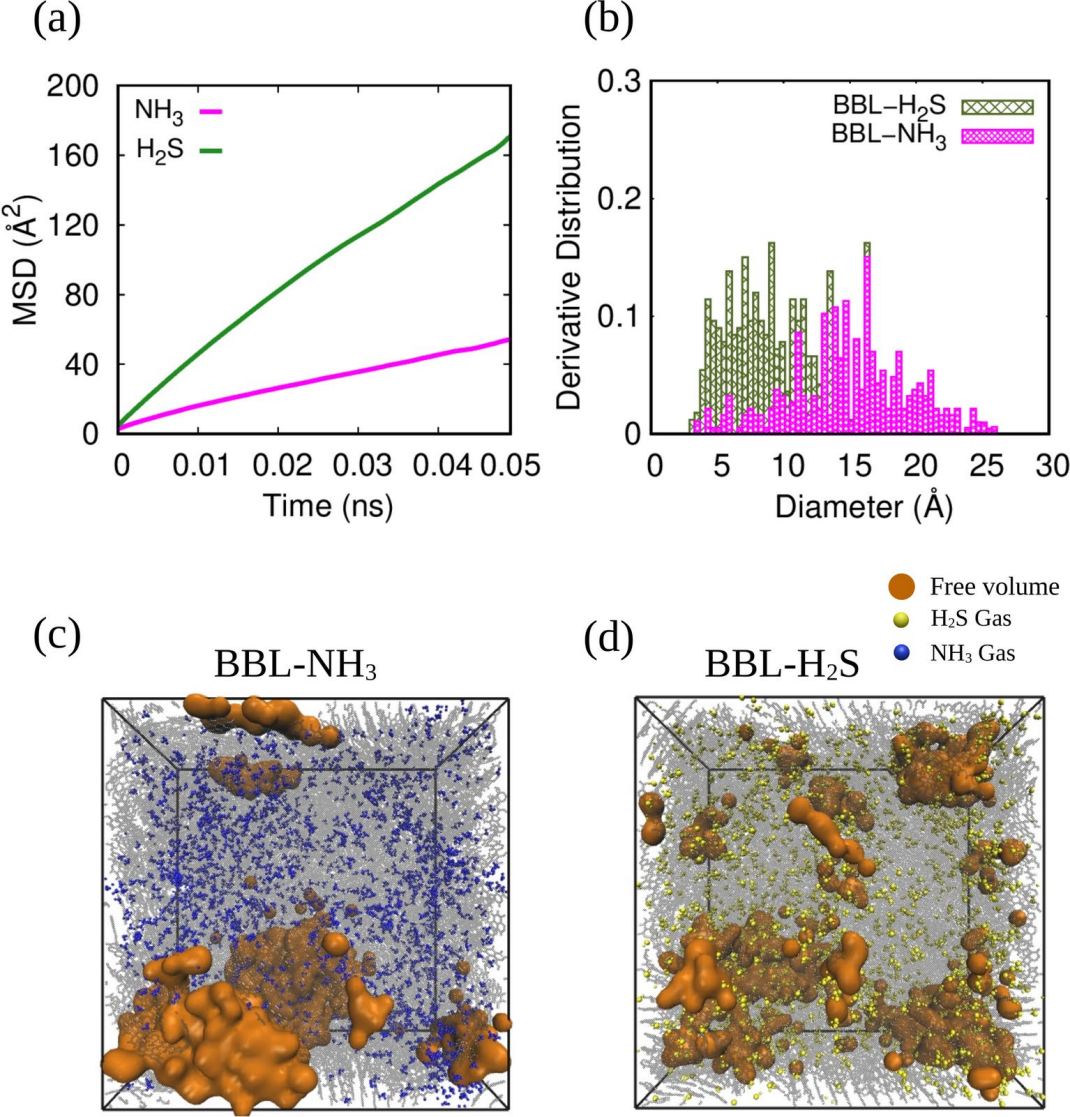
(**a**) Mean square displacement of NH$_{3}$ and H$_{2}$S in BBL film, (**b**) pore size distribution of H$_{2}$S and NH$_{3}$ loaded BBL films. Free volume distribution in (**c**) BBL-NH$_{3}$ and (**d**) BBL-H$_{2}$S systems. BBL chains, NH$_{3}$, H$_{2}$S, and pores are shown in black, blue, yellow, and orange, respectively.

Next, we analyzed the diffusion of the gases into the BBL film by calculating their diffusion coefficient to understand the sensitivity of the material to a specific analyte gas using MD simulations. The diffusivity of gas molecules is crucial in gas sensing applications since a high diffusivity limits the interaction time with the thin film, and, on the other hand, a low diffusivity limits the movement within the film, thus affecting the sensor’s sensitivity^74^. To calculate the diffusion coefficient (*D*), we calculated the mean square displacement (*MSD*), see Fig. 8a and used the Einstein relation to calculate the diffusion coefficient^75^ (*D*), tabulated in Table 1.

1 $$\begin{aligned} \textit{D}=\frac{MSD}{6t} \end{aligned}$$

We observed significantly larger *MSD* and *D* for H$_{2}$S gas than for NH$_{3}$. The observed diffusion coefficients and van der Waals interaction energies calculated by MD simulations align well with the results obtained from our DFT calculations. The higher gas-polymer interaction energy for NH$_{3}$ molecules, calculated from both DFT calculations and MD simulations, allows them to align closer to the polymer chains and, in turn, causes a reduction in the diffusion rate. On the other hand, due to weaker binding energy for H$_{2}$S, they diffuse faster than NH$_{3}$ molecules. Note, for microporous materials with pore diameters ranging from 10 to 20 Å, the Knudsen effect dominates diffusion, and the sensor sensitivity becomes inversely proportional to the diffusion coefficient of the gas molecules^74^. Hence, the lower MSD and *D* for NH$_{3}$ than H$_{2}$S indicates a better sensitivity of the BBL film for NH$_{3}$. We also compared our findings with past experimental results of NH$_{3}$ sensing using a p-type conducting polymer polyaniline (PANI)^59,76^. In particular, the calculated diffusion coefficient for ammonia in the BBL film, which is 0.96 $\times 10^{-7}$ cm$^{2}$s$^{-1}$, is lower than that in the PANI film, which is 6.7 $\times 10^{-7}$ cm$^{2}$sec$^{-1}$, as reported by Guo et al.^76^. It is worth noting that the diffusivity of NH$_{3}$ in the BBL film is 1.5–3% lower than the diffusivity of NH$_{3}$ in hydrochloric acid doped polyaniline (HCl-PANI) polymer film, while the interaction energy is 3–11% higher for the BBL film compared to HCl-PANI^59^, indicating a better performance of BBL than PANI in NH$_{3}$ sensing. Furthermore, PANI, being a p-type conducting polymer, was doped before exposure to NH$_{3}$ and upon NH$_{3}$ detection, the conductivity decreases, and the above-mentioned study showed that with increasing HCl dopant, the interaction energy towards NH$_{3}$ increases and diffusivity decreases. Therefore, the response depends on the doping concentration. In contrast, since BBL is an n-type polymer, the response should not depend on doping concentration, and the conductivity should continuously increase with NH$_{3}$ detection upon accepting the electrons from the reducing gas. This supports the notion that BBL would offer more excellent selectivity towards reducing gases than p-type conducting polymers.

Finally, we analyzed the free volume and the pore size distribution (PSD) of the loaded BBL film after exposure to the H$_{2}$S and NH$_{3}$. The pore size distributions of the gas-loaded BBL film are shown in Fig. 8b. Representative snapshots of the free volume of BBL-H$_{2}$S and BBL-NH$_{3}$ systems after the gas adsorption are shown in Fig. 8c, d. (A clearer representation of only the free volumes within BBL-H$_{2}$S and BBL-NH$_{3}$ systems without polymer chains and analyte gas molecules is shown in Supplementary Fig. S3). For the BBL-NH$_{3}$ system, i.e., upon NH$_{3}$ adsorption, the strong interaction energy between BBL and NH$_{3}$ leads to the compact packing of the gas molecules in the polymer matrix, and the free volumes that remain are mainly the bigger pores mainly close to the boundaries of the polymer matrix, as visible in Fig. 8c and Supplementary Fig. S3(a). Hence, the pore size distribution of the BBL film loaded with NH$_{3}$ shows pores of larger diameters, see Fig. 8b. On the contrary, in the case of the BBL-H$_{2}$S system, H$_{2}$S molecules are comparatively loosely packed throughout the BBL polymer matrix, resulting in the distribution of the pores with a smaller diameter compared to the BBL-NH$_{3}$ system, and the distributions of pores are not much being affected after gas adsorption with respect to the PSD of only BBL, see Fig. 1b and also not resulting in formation of the big pores in the boundaries of the BBL matrix (as shown in the PSD of BBL-H$_{2}$S system in Fig. 8b). Finally, we note, H$_{2}$S exhibits higher diffusivity than NH$_{3}$, and BBL has a stronger affinity for NH$_{3}$ molecules, indicating that the sensing response for NH$_{3}$ will be better compared to H$_{2}$S without affecting the crystalline nature of the film.

## Conclusions

In this study, we have gained valuable insight into the remarkable properties of an n-type conducting polymer, ladder-type polybenzimidazole benzophenanthroline (BBL), and its potential applications in gas sensing for detecting reducing gases, H$_{2}$S, NH$_{3}$. BBL film maintains its crystallinity, defined as the presence of crystallites made of $\pi$-$\pi$ stacked chains, even upon gas exposure, which is crucial for its effectiveness as an active material in gas sensor devices, as the $\pi$-$\pi$ stacked chains provide the path of electron transport. In addition, the film remains chemically unaltered, indicating the effectiveness of repeatability. However, further research and exploration are necessary to reach a definitive conclusion in this field. From DFT calculations, we observe that the BBL polymer exhibits a higher magnitude of adsorption energy for the NH$_{3}$ gas molecules over H$_{2}$S and other gas molecules commonly present in the air, indicating BBL polymer is selective towards ammonia gas compared to hydrogen sulfide gas and other gases commonly present in the air, making it a promising material for NH$_{3}$ detection. Our results also reveal that BBL is more selective and sensitive towards NH$_{3}$ as compared to H$_{2}$S, as the interaction energy, magnitude of charge transfer is higher for NH$_{3}$ with a lower diffusion coefficient. This study reveals the potential of BBL to be used as an active material of NH$_{3}$ sensor. In general, the findings presented in this study contribute to the future design and optimization of gas sensors based on n-type conducting polymer, BBL, for detecting reducing gases, like H$_{2}$S and NH$_{3}$, ultimately leading to improved safety and detection of toxic gases in various industrial and environmental applications.

## Methods

### DFT study

**Fig. 9:**
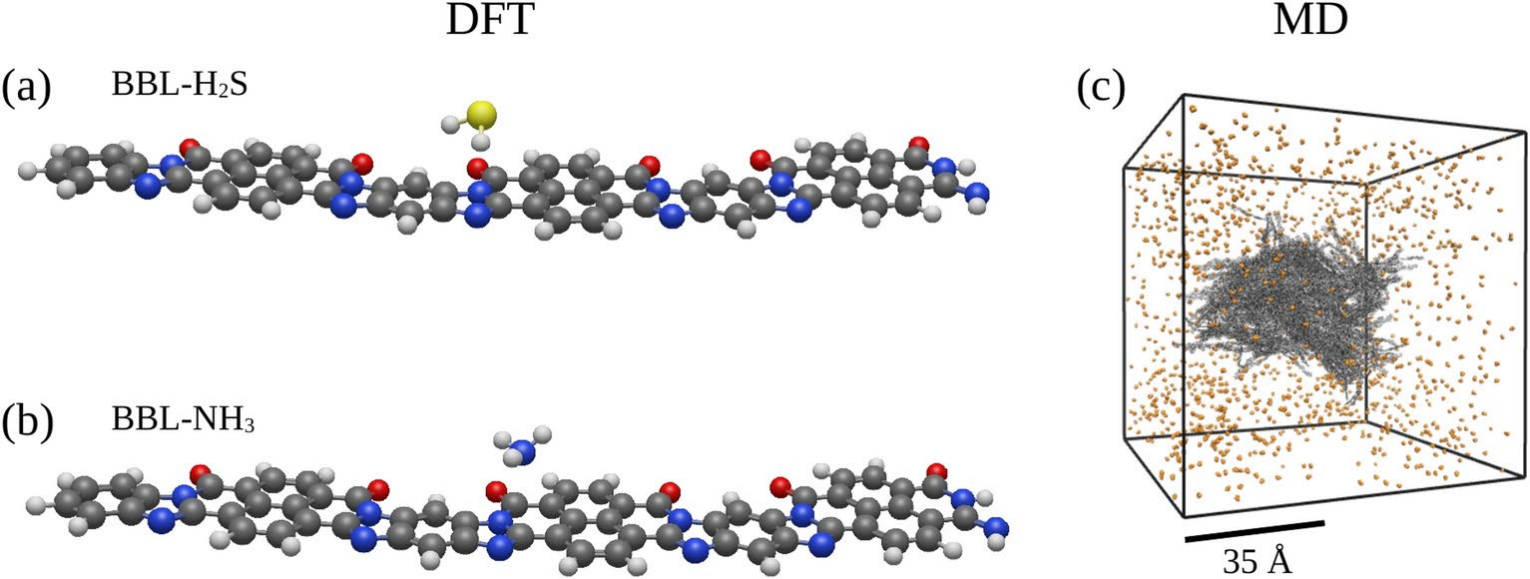
DFT optimized geometry structures of (**a**) BBL-H$_{2}$S and (**b**) BBL-NH$_{3}$ (a monomer of BBL polymer consisting of one benzene ring, one imidazole ring, one naphthalene ring, and one pyridine ring, respectively, one fused with the next). Carbon, nitrogen, oxygen, sulfur, and hydrogen are shown in grey, blue, red, yellow, and silver, respectively. (**c**) The BBL dry film, shown in grey color, kept in the center of the box surrounded by gas molecules represented in orange color.

We have investigated the interaction of H$_{2}$S and NH$_{3}$ gas molecules with a single BBL chain using density functional theory (DFT) in Gaussian 16^77^. We optimized the geometries of a single 3-mer BBL polymer chain, BBL in the presence of one H$_{2}$S molecule, and BBL in the presence of one NH$_{3}$ molecule, at the $\omega B97XD/6-31G(d)$^78^ level of DFT, without applying any constraints on the initial structures. Note, $\omega B97XD$^78^ is the range-separated hybrid functional, which includes the empirical atom-atom dispersion correction, which accounts for weaker London forces and improves accuracy in structural optimization and binding energy calculations. Fig. 9a and b show the DFT optimized structures of BBL-H$_{2}$S and BBL-NH$_{3}$. We have investigated the adsorption energy (E$_{ads}$), equilibrium adsorption distance (*d*), and the magnitude of charge transfer (CT) of H$_{2}$S and NH$_{3}$ gas molecules in the BBL polymer. The adsorption energies were calculated using the following formula.

2 $$\begin{aligned} E_{ads}=E_{BBL-H_{2}S/NH_{3}} - (E_{BBL}+E_{H_{2}S/NH_{3}} ) \end{aligned}$$

where, E$_{BBL}$, E$_{H_{2}S/NH_{3}}$ and E$_{BBL-H_{2}S/NH_{3}}$ are the energy of the BBL polymer, the energy of the isolated gas molecules and the total energy of BBL-gas systems, respectively.

The charge transfer analysis (Q$_{t}$) was done between the gas molecules and the BBL polymer using the following equation,

3 $$\begin{aligned} Q_{t} = Q_{a} - Q_{b} \end{aligned}$$

Where, Q$_{a}$ = Charge carried by gas molecules after the adsorption, Q$_{b}$ = Charge carried by gas molecules before the adsorption, Q$_{t}$ = Charge transfer. The charge carried by the gas molecules was calculated by the natural bond order (NBO) analysis using the keyword POP=NPA, i.e., the natural population analysis phase of NBO, where the atomic partial charges were obtained by summing over the natural atomic orbitals (NAOs). Here, the charge carried by gas molecules before the adsorption is considered null, i.e., the computation uses neutral molecules (BBL polymer chain and gas molecule)^79^. We calculated the density of states of all DFT-optimized systems using GaussSum software^80^.

### Preparation of active layer

MD simulations were performed in LAMMPS software^81^ using General AMBER Force Field (GAFF)^82^ generated with the moltemplate code^83^ to prepare a BBL dry film. We considered a BBL chain made of 10-unit monomers. 200 BBL chains were randomly packed in a cubical box of $18\times 18\times 16$ ${\rm nm}^{3}$ using Packmol software^84^ without any position overlap. The film was prepared using the melt-quench technique. The system was equilibrated in the *NPT* ensemble for $\sim$ 5 ns at 503 K temperature and 1 atm pressure. The system was then quenched to room temperature (303 K) at a rate of 4 K/100 ps and then equilibrated again at 303K and 1 atm for $\sim$ 10 ns. The Lennard-Jones interaction cutoff was set to 0.9nm. The K-scheme of the particle-particle-particle-mesh (PPPM) method was used to calculate the long-range Coulombic interaction with a 1.0 nm cutoff. More details on the film preparation can be found in our past study^85^.

To simulate the BBL- H$_{2}$S and BBL- NH$_{3}$ systems, we first placed the dry film of BBL in the center of the simulation box, and then surrounded it with 2000 gas molecules of either H$_{2}$S or NH$_{3}$ in a computational box as shown in Fig. 9c. The system was then equilibrated in an isothermal-isobaric (NPT) ensemble at a temperature of 303 K and a pressure of 1 atm for 10 ns. The partial charges on each atom of H$_{2}$S and NH$_{3}$ are calculated using the DFT calculation in the Gaussian package^86^ with the $\omega$b97xd functional and the 6-31g(d) basis set (Supplementary Fig. S16a). See Supplementary Information for detailed information regarding the simulation setup.

After equilibration, X-ray diffraction (XRD) patterns were simulated using LAMMPS^87^ with a wavelength of 1.54 Å to analyze and characterize the system’s morphology. Furthermore, we calculate the radial distribution function, *g(r)* to analyze the selectivity of gases to polymer and the mean square displacement (MSD)^88^ to calculate the diffusion coefficient (*D*) of two gases. The MSD was calculated as the average squared distance traveled by the gas molecules over time *t* relative to their initial positions using the following equation^89,90^.

4 $$\begin{aligned} MSD= \lim _{t \rightarrow \infty } \Bigl \langle |r_{i}(t)-r_{i}(0)|^{2}\Bigr \rangle \end{aligned}$$

where, $\bigl \langle .. \bigr \rangle$ represents ensemble average and $r_{i}(t)$ and $r_{i}(0)$ are the position of particles at t and $t_{0}$, respectively. The MSD as a function of time was fitted to a straight line ($MSD= 6Dt + C$), and the slope of the line was used to calculate diffusion coefficient *D*^89^. We have analyzed the distribution of voids and accessible sites for gas adsorption using the Zeo++ package^91^. The van der Waals and Coulombic interaction energies within the systems were computed using the LAMMPS package to understand the bulk pairwise interactions.

## Supplementary Information

Supplementary Information 1.

Supplementary Information 2.

## Data Availability

The datasets used and/or analysed during the current study are available from the corresponding author on reasonable request.

## References

[1] Preethichandra, D., Gholami, M. D., Izake, E. L., O’Mullane, A. P. & Sonar, P. Conducting polymer based ammonia and hydrogen sulfide chemical sensors and their suitability for detecting food spoilage. *Adv. Mater. Technol.* **8**, 2200841 (2023).

[2] Lambert, T. W., Goodwin, V. M., Stefani, D. & Strosher, L. Hydrogen sulfide (h2s) and sour gas effects on the eye. a historical perspective. *Sci. Total Env.* **367**, 1–22 (2006). 10.1016/j.scitotenv.2006.01.034 16650463

[3] Asghar, U. et al. Review on the progress in emission control technologies for the abatement of co2, sox and nox from fuel combustion. *J. Environ. Chem. Eng.* **9**, 106064 (2021).

[4] Pandey, J. S., Kumar, R. & Devotta, S. Health risks of no2, spm and so2 in delhi (india). *Atmos. Env.* **39**, 6868–6874 (2005).

[5] Rubright, S. L. M., Pearce, L. L. & Peterson, J. Environmental toxicology of hydrogen sulfide. *Nitric Oxide* **71**, 1–13 (2017). 29017846 10.1016/j.niox.2017.09.011 PMC5777517

[6] Farea, M. A. et al. Hazardous gases sensors based on conducting polymer composites. *Chem. Phys. Lett.* **776**, 138703 (2021).

[7] Guo, Z., Chen, G., Zeng, G., Liu, L. & Zhang, C. Metal oxides and metal salt nanostructures for hydrogen sulfide sensing: mechanism and sensing performance. *RSC Adv.* **5**, 54793–54805 (2015).

[8] Su, P.-G. & Peng, Y.-T. Fabrication of a room-temperature h2s gas sensor based on ppy/wo3 nanocomposite films by in-situ photopolymerization. *Sens. Actuat. B: Chem.* **193**, 637–643 (2014).

[9] Bai, S. et al. Polythiophene-wo3 hybrid architectures for low-temperature h2s detection. *Sens. Actuat. B: Chem.* **197**, 142–148 (2014).

[10] Beauchamp, R. et al. A critical review of the literature on hydrogen sulfide toxicity. *CRC Crit. Rev. Toxicol.* **13**, 25–97 (1984). 10.3109/10408448409029321 6378532

[11] Rubright, S. L. M., Pearce, L. L. & Peterson, J. Environmental toxicology of hydrogen sulfide. *Nitric oxide: Biol. Chem.* **71**, 1 (2017). 10.1016/j.niox.2017.09.011 PMC5777517 29017846

[12] Duc, C., Boukhenane, M.-L., Wojkiewicz, J.-L. & Redon, N. Hydrogen sulfide detection by sensors based on conductive polymers: a review. *Front. Mater.* **7**, 215 (2020).

[13] Timmer, B., Olthuis, W. & Van Den Berg, A. Ammonia sensors and their applications-a review. *Sens. Actuat. B: Chem.* **107**, 666–677 (2005).

[14] Mani, G. K. & Rayappan, J. B. B. A highly selective and wide range ammonia sensor-nanostructured zno: Co thin film. *Mater. Sci. Eng.: B* **191**, 41–50 (2015).

[15] Kumar, L., Rawal, I., Kaur, A. & Annapoorni, S. Flexible room temperature ammonia sensor based on polyaniline. *Sens. Actuat. B: Chem.* **240**, 408–416 (2017).

[16] Chen, T.-Y. et al. Characteristics of zno nanorods-based ammonia gas sensors with a cross-linked configuration. *Sens. Actuat. B: Chem.* **221**, 491–498 (2015).

[17] Choi, S.-J. et al. Selective detection of acetone and hydrogen sulfide for the diagnosis of diabetes and halitosis using sno2 nanofibers functionalized with reduced graphene oxide nanosheets. *ACS Appl. Mater. Interfaces* **6**, 2588–2597 (2014). 24456186 10.1021/am405088q

[18] Endre, Z. H. et al. Breath ammonia and trimethylamine allow real-time monitoring of haemodialysis efficacy. *Physiol. Meas.* **32**, 115 (2010). 21149927 10.1088/0967-3334/32/1/008

[19] Hubbard, T. W. & Putnam, D. L. Method for diagnosis of helicobacter pylori infection. US Patent 7,014,612 (2006).

[20] Rout, C. S., Hegde, M., Govindaraj, A. & Rao, C. Ammonia sensors based on metal oxide nanostructures. *Nanotechnology* **18**, 205504 (2007).

[21] Shankar, P. & Rayappan, J. B. B. Gas sensing mechanism of metal oxides: the role of ambient atmosphere, type of semiconductor and gases-a review. *Sci. Lett. J.* **4**, 126 (2015).

[22] Shaik, R., Kampara, R. K., Kumar, A., Sharma, C. S. & Kumar, M. Metal oxide nanofibers based chemiresistive h2s gas sensors. *Coordin. Chem. Rev.* **471**, 214752 (2022).

[23] Joshi, N. et al. A review on chemiresistive room temperature gas sensors based on metal oxide nanostructures, graphene and 2d transition metal dichalcogenides. *Microchim. Acta* **185**, 1–16 (2018). 10.1007/s00604-018-2750-5 29594538

[24] Korotcenkov, G. & Cho, B. Metal oxide composites in conductometric gas sensors: achievements and challenges. *Sens. Actuator.B: Chem.* **244**, 182–210 (2017).

[25] Liu, X., Zheng, W., Kumar, R., Kumar, M. & Zhang, J. Conducting polymer-based nanostructures for gas sensors. *Coordin. Chem. Rev.* **462**, 214517 (2022).

[26] Wong, Y. C., Ang, B. C., Haseeb, A., Baharuddin, A. A. & Wong, Y. H. Conducting polymers as chemiresistive gas sensing materials: a review. *J. Electrochem. Soc.* **167**, 037503 (2020).

[27] Verma, A., Gupta, R., Verma, A. S. & Kumar, T. A review of composite conducting polymer-based sensors for detection of industrial waste gases. *Sens. Actuat. Rep.* **2023**, 100143 (2023).

[28] Maksymiuk, K. Chemical reactivity of polypyrrole and its relevance to polypyrrole based electrochemical sensors. *Electroanalysis* **18**, 1537–1551 (2006).

[29] Rad, A. S., Nasimi, N., Jafari, M., Shabestari, D. S. & Gerami, E. Ab-initio study of interaction of some atmospheric gases (so2, nh3, h2o, co, ch4 and co2) with polypyrrole (3 ppy) gas sensor: Dft calculations. *Sens. Actuat. B Chem.* **220**, 641–651 (2015).

[30] Nicolas-Debarnot, D. & Poncin-Epaillard, F. Polyaniline as a new sensitive layer for gas sensors. *Anal. Chim. Acta* **475**, 1–15 (2003).

[31] Persaud, K. C. Polymers for chemical sensing. *Mater. Today* **8**, 38–44 (2005).

[32] Mazzotta, E., Rella, S., Turco, A. & Malitesta, C. Xps in development of chemical sensors. *RSC Adv.* **5**, 83164–83186 (2015).

[33] Fratoddi, I., Venditti, I., Cametti, C. & Russo, M. V. Chemiresistive polyaniline-based gas sensors: a mini review. *Sens. Actuator. B: Chem.* **220**, 534–548 (2015).

[34] Jain, A. et al. Fabrication of polypyrrole gas sensor for detection of nh3 using an oxidizing agent and pyrrole combinations: studies and characterizations. *Heliyon* (2023). 10.1016/j.heliyon.2023.e17611 PMC10338976 37455973

[35] Tan, H. et al. High-performance flexible gas sensors based on layer-by-layer assembled polythiophene thin films. *Chem. Mater.* **33**, 7785–7794 (2021).

[36] Farea, M. O. et al. High performance of carbon monoxide gas sensor based on a novel pedot: Pss/ppa nanocomposite. *ACS omega* **7**, 22492–22499 (2022). 35811925 10.1021/acsomega.2c01664 PMC9260891

[37] Wong, Y. C., Ang, B. C., Haseeb, A., Baharuddin, A. A. & Wong, Y. H. Conducting polymers as chemiresistive gas sensing materials: a review. *J. Electrochem. Soc.* **167**, 037503 (2020).

[38] Abdali, H., Heli, B. & Ajji, A. Stable and sensitive amino-functionalized graphene/polyaniline nanofiber composites for room-temperature carbon dioxide sensing. *RSC Adv.* **9**, 41240–41247 (2019). 35540051 10.1039/c9ra06223h PMC9076375

[39] Wang, Y., Liu, A., Han, Y. & Li, T. Sensors based on conductive polymers and their composites: a review. *Polym. Int.* **69**, 7–17 (2020).

[40] Shariq, M. U., Husain, A., Khan, M. & Ahmad, A. Synthesis and characterization of polypyrrole/molybdenum oxide composite for ammonia vapour sensing at room temperature. *Polym. Polym. Compos.* **29**, S989–S999 (2021).

[41] Yan, Y. et al. Conducting polymer-inorganic nanocomposite-based gas sensors: a review. *Sci. Technol. Adv. Mater.* **21**, 768–786 (2020). 10.1080/14686996.2020.1820845 PMC7801028 33488297

[42] Madgula, K. & Shubha, L. Conducting polymer nanocomposite-based gas sensors. *Funct. Nanomater. Adv. Gas Sens. Technol.* **2020**, 399–431 (2020).

[43] Savin, M. et al. Resistive chemosensors for the detection of co based on conducting polymers and carbon nanocomposites: a review. *Molecules* **27**, 821 (2022). 35164084 10.3390/molecules27030821 PMC8838520

[44] Choi, J., Song, H., Kim, N. & Kim, F. S. Development of n-type polymer semiconductors for organic field-effect transistors. *Semicond. Sci. Technol.* **30**, 064002 (2015).

[45] Han, Y. et al. Anion-induced n-doping of naphthalenediimide polymer semiconductor in organic thin-film transistors. *npj Flexible Electron.* **2**, 11 (2018).

[46] Song, H. et al. Crystal structure and thin film morphology of bbl ladder polymer. *Synth. Metals* **69**, 533–535 (1995).

[47] Kim, O.-K. Ladder polymers as new polymeric conductors. *Mol. Crystals Liquid Crystals* **105**, 161–173 (1984).

[48] Jenekhe, S. A. & Tibbetts, S. J. Ion implantation doping and electrical properties of high-temperature ladder polymers. *J. Polym. Sci. Part B: Polym. Phys.* **26**, 201–209 (1988).

[49] Arnold, F. & Van Deusen, R. Unusual film-forming properties of aromatic heterocyclic ladder polymers. *J. Appl. Polym. Sci.* **15**, 2035–2047 (1971).

[50] Vagin, M. et al. Negatively-doped conducting polymers for oxygen reduction reaction. *Adv. Energy Mater.* **11**, 2002664 (2021).

[51] Babel, A. & Jenekhe, S. A. High electron mobility in ladder polymer field-effect transistors. *J. Am. Chem. Soc.* **125**, 13656–13657 (2003). 14599192 10.1021/ja0371810

[52] Duc, C., Boukhenane, M.-L., Wojkiewicz, J.-L. & Redon, N. Hydrogen sulfide detection by sensors based on conductive polymers: a review. *Front. Mater.* **7**, 215 (2020).

[53] Zhou, G., Cao, Z., Liu, Y., Zheng, H. & Xu, K. Highly sensitive and stable glucose sensing using n-type conducting polymer based organic electrochemical transistor. *J. Electroanal. Chem.* **952**, 117961 (2024).

[54] Oliveira, G. P., Barboza, B. H. & Batagin-Neto, A. Polyaniline-based gas sensors: Dft study on the effect of side groups. *Comput. Theor. Chem.* **1207**, 113526 (2022).

[55] Azak, H., Gorgul, R., Tekin, B. & Yildiz, M. Calculation of conductive polymer-based so2 and so3 gas sensor mechanisms by using the dft method. *J. Mol. Model.* **25**, 367 (2019). 31776788 10.1007/s00894-019-4219-9

[56] Boboriko, N. E. & Dzichenka, Y. U. Molecular dynamics simulation as a tool for prediction of the properties of tio2 and tio2: Moo3 based chemical gas sensors. *J. Alloys Compounds* **855**, 157490 (2021).

[57] Huang, S. et al. Highly sensitive room temperature ammonia gas sensor using pristine graphene: the role of biocompatible stabilizer. *Carbon* **173**, 262–270 (2021).

[58] Sinha, M., Rana, M. K. & Panda, S. Templated growth of polyaniline for enhanced gas sensing response in flexible sensors: experiments and simulations. *Flexible Printed Electron.* **6**, 035007 (2021).

[59] Pang, Z., Yildirim, E., Pasquinelli, M. A. & Wei, Q. Ammonia sensing performance of polyaniline-coated polyamide 6 nanofibers. *ACS omega* **6**, 8950–8957 (2021). 33842765 10.1021/acsomega.0c06272 PMC8028015

[60] Liu, P. & Chen, G. Chapter one—general introduction to porous materials. In *Porous Materials* (eds Liu, P. & Chen, G.) 1–20 (Butterworth-Heinemann, 2014).

[61] Zhang, Y. et al. Ultrasensitive flexible nh3 gas sensor based on polyaniline/srge4o9 nanocomposite with ppt-level detection ability at room temperature. *Sens. Actuator. B: Chem.* **319**, 128293 (2020).

[62] Khurshid, F., Jeyavelan, M., Hussain, T., Hudson, M. S. L. & Nagarajan, S. Ammonia gas adsorption study on graphene oxide based sensing device under different humidity conditions. *Mater. Chem. Phys.* **242**, 122485 (2020).

[63] Sharma, A., Malani, A., Medhekar, N. V. & Babarao, R. Co 2 adsorption and separation in covalent organic frameworks with interlayer slipping. *CrystEngComm* **19**, 6950–6963 (2017).

[64] Yao, Z.-F., Wang, J.-Y. & Pei, J. Control of π-π stacking via crystal engineering in organic conjugated small molecule crystals. *Crystal Growth Design* **18**, 7–15 (2018).

[65] Coropceanu, V. et al. Charge transport in organic semiconductors. *Chem. Rev.* **107**, 926–952 (2007). 17378615 10.1021/cr050140x

[66] Petsagkourakis, I. et al. Structurally-driven enhancement of thermoelectric properties within poly (3, 4-ethylenedioxythiophene) thin films. *Sci. Rep.* **6**, 30501 (2016). 27470637 10.1038/srep30501 PMC4965772

[67] Mohammadi, M. D., Salih, I. H. & Abdullah, H. Y. The adsorption of chlorofluoromethane on pristine and ge-doped silicon carbide nanotube: a pbc-dft, nbo, and qtaim study. *Mol. Simul.* **46**, 1405–1416 (2020). 10.1007/s00894-020-04556-5 32980919

[68] Rad, A. S., Nasimi, N., Jafari, M., Shabestari, D. S. & Gerami, E. Ab-initio study of interaction of some atmospheric gases (so2, nh3, h2o, co, ch4 and co2) with polypyrrole (3 ppy) gas sensor: Dft calculations. *Sens. Actuat. B: Chem.* **220**, 641–651 (2015).

[69] Anasthasiya, A. N. A., Rai, P. & Jeyaprakash, B. Understanding ammonia adsorption and charge transfer process on zno using experimental and dft approach. *Mater. Chem. Phys.* **214**, 540–547 (2018).

[70] Sun, H. & Autschbach, J. Electronic energy gaps for π-conjugated oligomers and polymers calculated with density functional theory. *J. Chem. Theory Comput.* **10**, 1035–1047 (2014). 26580181 10.1021/ct4009975

[71] Ghosh, S., Gueskine, V., Berggren, M. & Zozoulenko, I. V. Electronic structures and optical absorption of n-type conducting polymers at different doping levels. *J. Phys. Chem. C* **123**, 15467–15476 (2019).

[72] Ghosh, S., Rolland, N. & Zozoulenko, I. Electronic structure, optical properties, morphology and charge transport in naphthalenediimide (ndi)-based n-type copolymer with altered π-conjugation: a theoretical perspective. *Appl. Phys. Lett.* **118**, 896 (2021).

[73] Esmaeili, C. et al. Preparation and characterisation of nh3 gas sensor based on pani/fe-doped ceo2 nanocomposite. *Heliyon* **10**, 896 (2024). 10.1016/j.heliyon.2024.e34801 PMC11336279 39170534

[74] Sakai, G., Matsunaga, N., Shimanoe, K. & Yamazoe, N. Theory of gas-diffusion controlled sensitivity for thin film semiconductor gas sensor. *Sens. Actuator. B: Chem.* **80**, 125–131 (2001).

[75] Sedghamiz, T., Mehandzhiyski, A. Y., Modarresi, M., Linares, M. & Zozoulenko, I. What can we learn about pedot:pss morphology from molecular dynamics simulations of ionic diffusion?. *Chem. Mater.* **35**, 5512–5523 (2023).

[76] Guo, Z., Liao, N., Zhang, M. & Feng, A. Enhanced gas sensing performance of polyaniline incorporated with graphene: a first-principles study. *Phys. Lett. A* **383**, 2751–2754 (2019).

[77] Frisch, M. e. et al. Gaussian 16, revision c. 01 (2016).

[78] Lin, Y.-S., Li, G.-D., Mao, S.-P. & Chai, J.-D. Long-range corrected hybrid density functionals with improved dispersion corrections. *J. Chem. Theory Comput.* **9**, 263–272 (2013). 26589028 10.1021/ct300715s

[79] Wei, H., Gui, Y., Kang, J., Wang, W. & Tang, C. A dft study on the adsorption of h2s and so2 on ni doped mos2 monolayer. *Nanomaterials* **8**, 646 (2018). 30135410 10.3390/nano8090646 PMC6164490

[80] O’boyle, N. M., Tenderholt, A. L. & Langner, K. M. Cclib: a library for package-independent computational chemistry algorithms. *J. Comput. Chem.* **29**, 839–845 (2008). 10.1002/jcc.20823 17849392

[81] Thompson, A. P. et al. Lammps-a flexible simulation tool for particle-based materials modeling at the atomic, meso, and continuum scales. *Comput. Phys. Commun.* **271**, 108171 (2022).

[82] Wang, J., Wolf, R. M., Caldwell, J. W., Kollman, P. A. & Case, D. A. Development and testing of a general amber force field. *J. Comput. Chem.* **25**, 1157–1174 (2004). 15116359 10.1002/jcc.20035

[83] Jewett, A. I. et al. Moltemplate: a tool for coarse-grained modeling of complex biological matter and soft condensed matter physics. *J. Mol. Biol.* **433**, 166841 (2021). 33539886 10.1016/j.jmb.2021.166841 PMC8119336

[84] Martínez, L., Andrade, R., Birgin, E. G. & Martínez, J. M. Packmol: a package for building initial configurations for molecular dynamics simulations. *J. Comput. Chem.* **30**, 2157–2164 (2009). 19229944 10.1002/jcc.21224

[85] Sunny, S., Shah, S., Garg, M., Zozoulenko, I. & Ghosh, S. Microscopic insights of electrochemical switching of poly(benzimidazobenzophenanthroline)(bbl) thin film: a molecular dynamics study. *Macromolecules* (2024).

[86] Ghosh, S., Berggren, M. & Zozoulenko, I. Electronic structures and optical properties of p-type/n-type polymer blends: density functional theory study. *J. Phys. Chem. C Nanomater. Interfaces* **124**, 9203–9214 (2020).

[87] Coleman, S. P., Sichani, M. M. & Spearot, D. E. A computational algorithm to produce virtual x-ray and electron diffraction patterns from atomistic simulations. *JOM* **1989**(66), 408–416 (2014).

[88] Franco-Gonzalez, J. F. & Zozoulenko, I. V. Molecular dynamics study of morphology of doped pedot: From solution to dry phase. *J. Phys. Chem. B* **121**, 4299–4307 (2017). 28380297 10.1021/acs.jpcb.7b01510

[89] Pang, Z., Yildirim, E., Pasquinelli, M. A. & Wei, Q. Ammonia sensing performance of polyaniline-coated polyamide 6 nanofibers. *ACS Omega* **6**, 8950–8957 (2021). 33842765 10.1021/acsomega.0c06272 PMC8028015

[90] Garg, M. & Zozoulenko, I. Ion diffusion through nanocellulose membranes: molecular dynamics study. *ACS Appl. Bio Mater.* **4**, 8301–8308 (2021). 35005924 10.1021/acsabm.1c00829

[91] Willems, T. F., Rycroft, C. H., Kazi, M., Meza, J. C. & Haranczyk, M. Algorithms and tools for high-throughput geometry-based analysis of crystalline porous materials. *Micropor. Mesopor. Mater.* **149**, 134–141 (2012).

