# Supplementary material for: Exploring an n-type conducting polymer (BBL) as a potential gas sensing material for NH3 and H2S detection
Source: Sci Rep. 2025 Mar 27;15:10623. doi: 10.1038/s41598-025-93977-4 (PMC11950291; doi:10.1038/s41598-025-93977-4)
Supplement: Supplementary file 1 — Supplementary Information 1. [file 41598_2025_93977_MOESM1_ESM.pdf]

Sl No	Atom	Charge						
1	C	-0.157978	53	C	0.326868	105	C	0.408428
2	C	0.461155	54	C	0.618013	106	N	-0.593003
3	C	-0.120608	55	C	0.121831	107	C	-0.056622
4	C	-0.181202	56	N	-0.480498	108	C	0.105772
5	C	0.524209	57	O	-0.521709	109	C	-0.180429
6	C	-0.519426	58	N	-0.530879	110	H	0.1433
7	N	-0.096251	59	H	0.184356	111	C	-0.216543
8	N	-0.608498	60	H	0.234106	112	C	0.137653
9	C	0.45188	61	H	0.119995	113	C	-0.05321
10	C	-0.192411	62	H	0.188651	114	C	0.590703
11	C	0.606427	63	H	0.120829	115	O	-0.469881
12	O	-0.471057	64	H	0.185079	116	C	-0.222662
13	C	-0.230378	65	C	-0.22319	117	C	-0.035984
14	C	0.126598	66	C	-0.124982	118	H	0.136084
15	C	0.154336	67	C	0.543433	119	C	0.462423
16	C	-0.041861	68	C	-0.15695	120	N	-0.081777
17	C	-0.064391	69	C	-0.556788	121	C	-0.162035
18	C	-0.215396	70	C	0.506741	122	C	-0.115599
19	C	-0.060039	71	C	0.596176	123	N	-0.578476
20	C	-0.04022	72	O	-0.472252	124	H	0.130752
21	C	-0.250678	73	C	-0.066227	125	C	0.445148
22	C	0.591517	74	C	-0.20365	126	H	0.193306
23	C	0.50212	75	C	0.443788	127	O	-0.466477
24	N	-0.120066	76	N	-0.093352	128	C	-0.197205
25	O	-0.467237	77	C	-0.047376	129	C	0.586539
26	N	-0.580741	78	C	0.111293	130	C	-0.486976
27	H	0.19238	79	C	-0.190974	131	C	0.519642
28	H	0.240352	80	N	-0.592904	132	N	-0.081327
29	H	0.138199	81	C	-0.21836	133	C	-0.060424
30	H	0.144766	82	C	0.145911	134	C	-0.195493
31	H	0.141605	83	C	-0.057334	135	H	0.141723
32	H	0.134709	84	C	0.589262	136	C	0.442645
33	C	-0.126993	85	C	-0.231848	137	C	-0.054396
34	C	0.419487	86	H	0.19711	138	N	-0.603813
35	C	-0.098088	87	C	-0.035747	139	C	0.095815
36	C	-0.262738	88	C	0.465908	140	H	0.232031
37	C	0.559397	89	O	-0.467987	141	C	-0.179371
38	C	-0.501287	90	N	-0.086329	142	C	-0.231624
39	N	0.023508	91	H	0.24799	143	H	0.143025
40	N	-0.632397	92	H	0.144681	144	C	0.166849
41	C	0.438532	93	N	-0.581259	145	C	-0.06485
42	C	-0.349925	94	H	0.14093	146	C	0.611115
43	C	0.55217	95	H	0.13844	147	O	-0.472871
44	O	-0.464984	96	H	0.132839	148	C	-0.253247
45	C	-0.324057	97	H	0.3688	149	C	-0.017493
46	C	0.299565	98	H	0.325965	150	C	0.487443
47	C	-0.175272	99	O	-0.462363	151	N	-0.12218
48	C	0.103778	100	C	0.559945	152	H	0.138932
49	C	-0.298358	101	N	-0.023991	153	C	-0.126953
50	C	0.073426	102	C	-0.057077	154	C	-0.143802
51	C	0.147496	103	C	-0.193059	155	N	-0.570301
52	C	-0.428283	104	H	0.141929	156	C	0.411647

157	H	0.124812	210	C	-0.226032	263	C	0.474495
158	O	-0.471792	211	C	0.609519	264	N	-0.575502
159	H	0.199866	212	H	0.142154	265	C	-0.055853
160	C	-0.15713	213	O	-0.470393	266	C	0.148651
161	C	0.619598	214	H	0.12967	267	C	-0.232428
162	C	-0.451192	215	C	0.478701	268	H	0.143172
163	C	0.483689	216	N	-0.120569	269	C	-0.201316
164	N	-0.120567	217	C	-0.160937	270	H	0.234489
165	C	-0.042998	218	N	-0.599079	271	C	0.108094
166	C	-0.244838	219	C	-0.154314	272	C	-0.027236
167	C	0.472027	220	C	0.492966	273	C	0.590168
168	H	0.138973	221	H	0.136407	274	C	-0.194622
169	N	-0.596269	222	C	-0.088917	275	C	-0.064798
170	C	-0.073501	223	O	-0.472009	276	H	0.140239
171	C	0.166314	224	C	-0.474238	277	O	-0.467912
172	H	0.224562	225	C	0.606671	278	C	0.460604
173	C	-0.229278	226	C	0.400769	279	N	-0.086812
174	C	-0.178438	227	H	0.200138	280	H	0.129399
175	C	0.097398	228	N	-0.12837	281	C	-0.208499
176	C	-0.039554	229	C	-0.0568	282	N	-0.605086
177	H	0.145265	230	C	-0.223306	283	C	-0.096499
178	C	0.562711	231	C	0.458814	284	C	0.532125
179	C	-0.201528	232	N	-0.555945	285	H	0.140499
180	O	-0.458676	233	C	-0.048069	286	C	-0.537227
181	C	-0.041648	234	C	0.141542	287	C	-0.184661
182	H	0.132559	235	C	-0.212369	288	O	-0.469058
183	C	0.445707	236	H	0.142312	289	H	0.189684
184	N	-0.072779	237	H	0.232196	290	C	0.49014
185	C	-0.166792	238	C	-0.219782	291	C	0.572161
186	N	-0.569802	239	C	0.123734	292	N	-0.051042
187	C	-0.117506	240	C	-0.048161	293	C	0.416478
188	H	0.129812	241	C	0.601341	294	N	-0.575524
189	C	0.451149	242	C	-0.204669	295	C	-0.209394
190	H	0.185529	243	O	-0.46882	296	H	0.242435
191	C	-0.184426	244	C	-0.046067	297	C	-0.036611
192	C	-0.471734	245	H	0.137493	298	C	-0.190078
193	C	0.478166	246	C	0.454703	299	C	0.117995
194	H	0.226487	247	N	-0.094395	300	H	0.137965
195	O	-0.462405	248	C	-0.180795	301	C	-0.088991
196	C	0.569713	249	C	-0.126294	302	C	-0.038609
197	N	-0.043104	250	H	0.131793	303	C	0.121735
198	C	0.405693	251	N	-0.597961	304	C	-0.194422
199	N	-0.568158	252	C	0.507824	305	C	-0.066894
200	C	-0.209189	253	C	-0.13571	306	C	-0.203586
201	C	-0.048642	254	O	-0.471666	307	H	0.130005
202	C	-0.182882	255	C	-0.495722	308	H	0.15006
203	C	0.113075	256	C	0.600365	309	C	0.644821
204	C	-0.064365	257	H	0.13691	310	O	-0.480709
205	C	-0.044278	258	C	0.43905	311	C	0.509234
206	H	0.140956	259	H	0.194881	312	N	-0.241538
207	C	0.143869	260	N	-0.112876	313	H	0.143187
208	C	-0.21735	261	C	-0.05709	314	N	-0.617987
209	C	-0.044407	262	C	-0.220029	315	C	0.010049

316	C	0.451576
317	C	-0.179452
318	C	-0.331554
319	H	0.170464
320	C	-0.181182
321	C	-0.098091
322	H	0.189334
323	H	0.14984
324	H	0.136274
