# Supplementary material for: Exploring an n-type conducting polymer (BBL) as a potential gas sensing material for NH3 and H2S detection
Source: Sci Rep. 2025 Mar 27;15:10623. doi: 10.1038/s41598-025-93977-4 (PMC11950291; doi:10.1038/s41598-025-93977-4)
Supplement: Supplementary file 2 — Supplementary Information 2. [file 41598_2025_93977_MOESM2_ESM.pdf]

Supplementary Information of

Exploring an N-Type Conducting Polymer (BBL) as a Potential Gas Sensing Material for NH_3 and H_2S Detection

Sonu Sunny[1], Sushri Soumya Jena[1], Shivam Shah[1], Bhavika Gopalani[1], Arnab Hazra[2], Mohit Garg[1], and Sarbani Ghosh[1,*]

[1]Department of Chemical Engineering, Birla Institute of Technology and Science (BITS), Pilani Campus, Vidya Vihar, Pilani, 333031, Rajasthan, India
[2]Department of Electrical and Electronics Engineering, Birla Institute of Technology and Science (BITS), Pilani Campus, Vidya Vihar, Pilani, 333031, Rajasthan, India
[*]sarbani.ghosh@pilani.bits-pilani.ac.in

Supplementary Information

S1 Morphology Analysis

S1.1 XRD Characterization

We have characterized the crystallinity of pristine BBL experimentally through XRD and compared with MD simulated XRD of the pristine BBL, see fig. S1. For experimental XRD, we observed peaks at 2θ=11.1°($d = n\lambda/2d\sin\theta = 8.33$Å) and 26.42°(d=3.36Å). The peak at 2θ= 10.6°corresponds to lamellar stacking, whereas the peak at 2θ= 26.42°corresponds to the $\pi-\pi$ stacking present in the polymer system. In the case of simulated XRD, we observed peaks at 2θ=10.55°(d=8.37Å) and 2θ=26.25°(d=3.39Å) corresponding to lamellar and $\pi-\pi$ stacking peaks, respectively. Here, we have shown the corresponding snapshots of the lamellar and $\pi-\pi$ stackings, which are present in the polymer system.

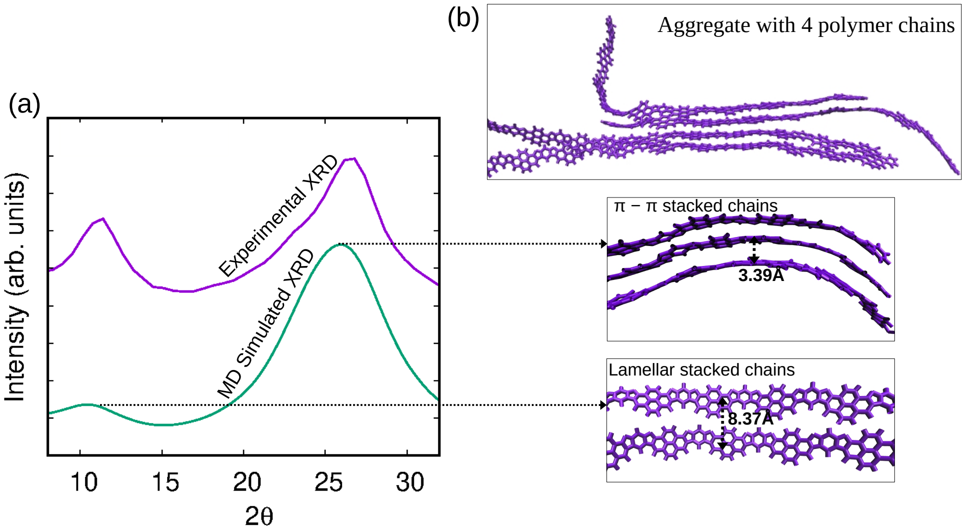

Figure S1. (a) Experimental and MD simulated XRD of BBL polymer, and (b) snapshot of an aggregate with four polymer chains.

S1.1.1 XRD Calculation in LAMMPS

To calculate the XRD of the MD simulated film, we have considered the last frame generated during the production stage of the MD simulation run. The XRD calculation in LAMMPS is based on an algorithm that computes the intensity of XRD on a mesh of reciprocal lattice points within the simulation domain or a manually defined grid(1). This process simulates the interaction of X-rays with the atomic structure, and we have used a wavelength of (λ=1.54Å) that corresponds to the experimental setup.

S1.1.2 Crystallite size

We calculated the crystallite size using the Scherrer equation and then divided it by stacking distance to calculate the number of polymer chains in the aggregates.

System	Crystallite Size (Å)	Number of Polymer chains
BBL Dry Film	13.7	$4.07 \approx 4$
BBL-H_2S	14.7	$4.33 \approx 4$
BBL-NH_3	14.2	$4.22 \approx 4$

Table S1. Calculated $\pi - \pi$ stack crystallite size and the approximate number of polymer chains in each crystallite from XRD data using the Scherrer equation.

S1.2 AA and AB $\pi - \pi$ - Stacks

π-stacking of two chains can show different arrangements based on the orientation of the chains, as shown in fig. S2. The similar charge distribution of the polymer chains, when aligned in the same direction, leads to electrostatic repulsion and results in a staggered arrangement which shows both longitudinal and transverse slipping in stacking distance (Δs),(2) which is more pronounced in AA stacking compared to AB stacking. In the MD-prepared film, we observed the formation of an eclipsed arrangement when two polymer chains are aligned in the opposite direction (AB) and a staggered arrangement when two polymer chains are aligned in the same direction (AA) due to the repulsion between like charges between atoms in the polymer chains(3), as shown in fig. S2. For the AA stacking, we found a slipping distance (Δs) in the range of ((4.6–5)-3.4=)∼1.2-1.6 Å and for AB stacking, we observed that the slipping distance is in the range of ((3.5–4.06)-3.4=) ∼0.1-0.7 Å, considering the $\pi - \pi$ stacking distance as 3.4 Å. In order to calculate the energy difference in both arrangements, we have optimized the structure for both the AB (eclipsed) and AA (staggered) $\pi - \pi$ stacked polymer chains using DFT. and found an insignificant difference in the optimized energy between the two configurations, as illustrated in fig. S2b, which says both structures can form in the film and that we observed in the MD-prepared film, as shown in fig. S2c. This is further quantified by calculating the distributions of interchain end-to-end distance of the polymers, which shows the formation of both AA and AB stacking in the bulk film, as shown in fig. S2d.

S1.3 PSD Analysis

To analyze the nanoporous structure, we conducted calculations for free volume, surface area, and pore size distribution (PSD) using the Zeo++ package. The free volume representation of BBL-H_2S and BBL-NH_3 systems without polymer chains and analyte gas molecules are given in fig. S3.

System	AV(m^3/g)	NAV (m^3/g)	ASA (m^2/g)	NASA (m^2/g)
Unloaded	6.68×10^{-8}	2.01×10^{-8}	332.339	264.353
BBL-H_2S	3.08×10^{-8}	0.55×10^{-8}	238.104	106.99
BBL-NH_3	6.31×10^{-8}	1.40×10^{-8}	213.089	239.253

Table S2. Calculated available volume, AV (m^3/g), non available volume, NAV (m^3/g), available surface area, ASA (m^2/g) and non available surface, NASA area (m^2/g) of unloaded, and NH_3 and H_2S loaded BBL.

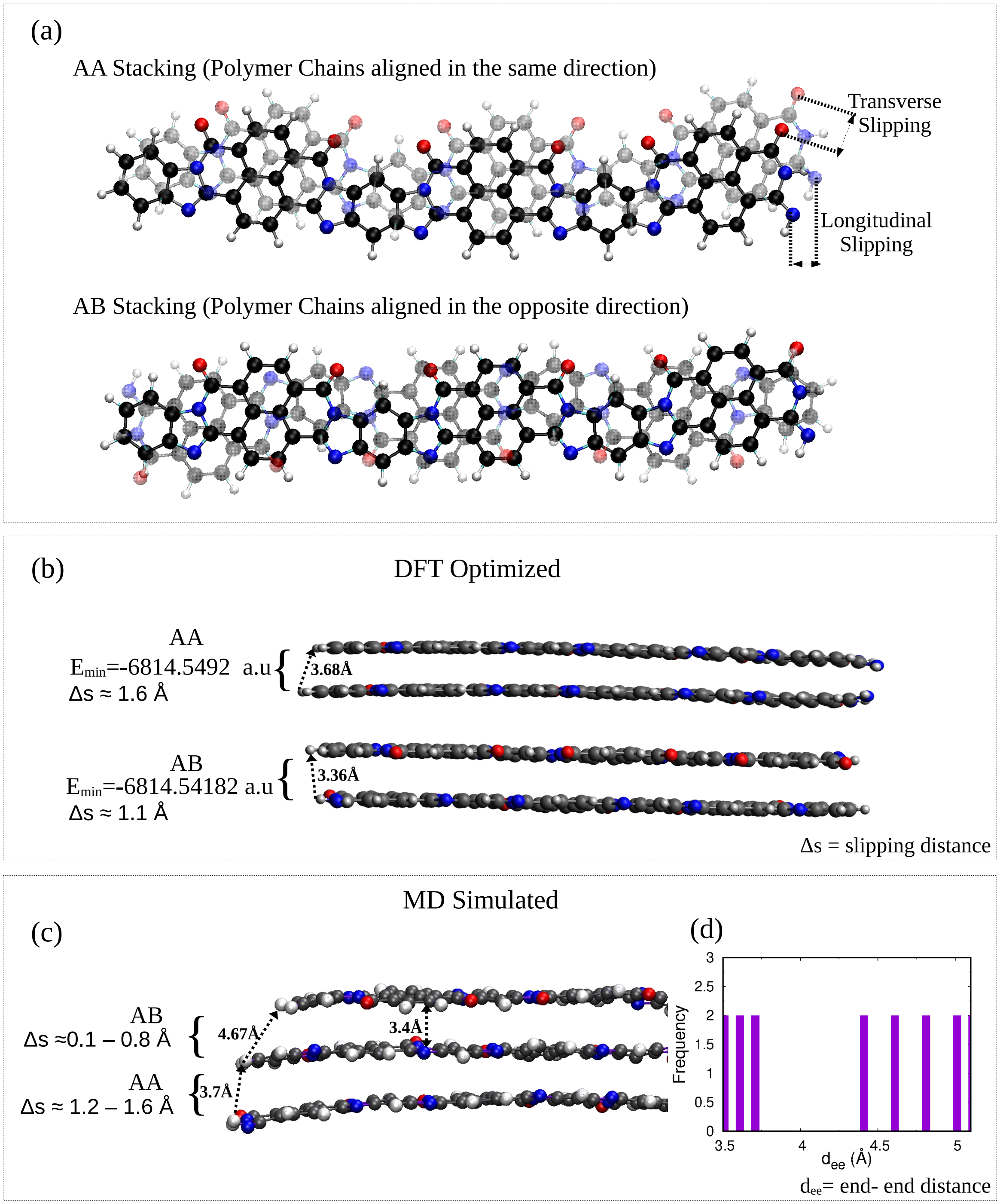

Figure S2. (a) Schematics of AB and AA stacking in stacked BBL trimers, (b) AB and AA stacking in stacked polymer chains obtained from DFT with corresponding slipping distance, (c) AB and AA stacking in stacked polymer chains obtained from MD simulations with corresponding slipping distance, and (d) the frequency distribution of end to end distance (d_{ee}) between stacked polymer chains.

S1.4 Swelling

We have calculated the % swelling of the loaded film compared to the unloaded film and found, for H_2S and NH_3 loaded film, the % *swelling* is $\sim 5.6\%$ and $\sim 5.36\%$, respectively.

$$\% \text{ swelling} = \frac{\text{Final volume} - \text{Initial volume}}{\text{Initial volume}} \times 100 \tag{1}$$

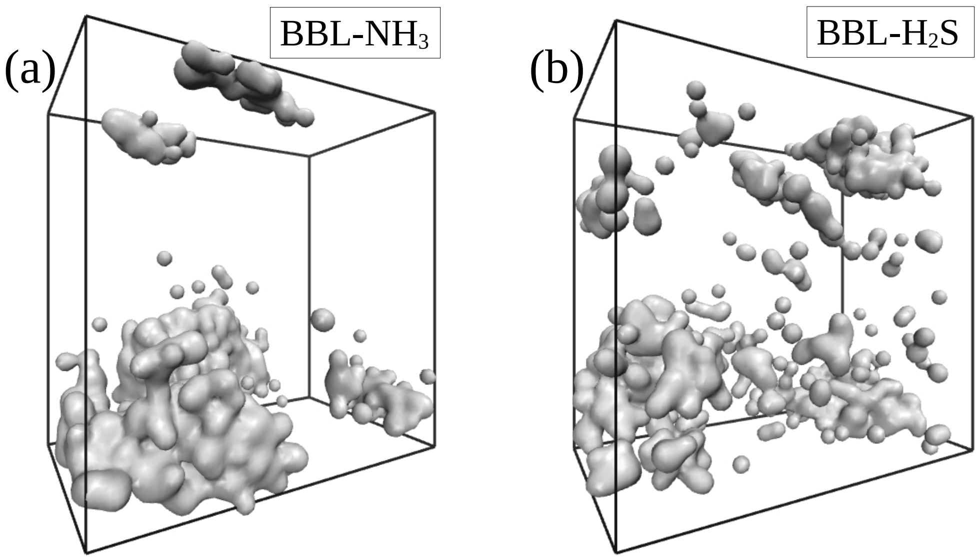

Figure S3. (a) Free volume distribution of (a) BBL-NH_3 and (b) BBL-H_2S systems.

S2 FTIR Characterization

In order to understand the structural and chemical changes before and after the gas adsorption, we performed an FTIR analysis of the sample. The FTIR spectra of the sample before and after gas adsorption in the range of 2000 to 600cm^{-1} are shown in Figure S4. We observed the C=O stretching band at 1700cm^{-1}, the C=C stretching band at 1500cm^{-1}, the C=N and C-N stretching in the range of 1400cm^{-1} to 1300cm^{-1} and finally the aromatic C-H bending at 995cm^{-1} (4). There is no appreciable change in the spectra of pristine BBL, indicating that the neutral BBL has not undergone any structural or chemical changes even after the gas adsorption. This underscores the potential of BBL polymer to act as a promising active material for gas sensing applications. The FTIR spectra were measured in transmission mode with Perkin Elmer Spectrum Version Frontier FT-IR, averaging 20 scans with a resolution of 4 cm^{-1}.

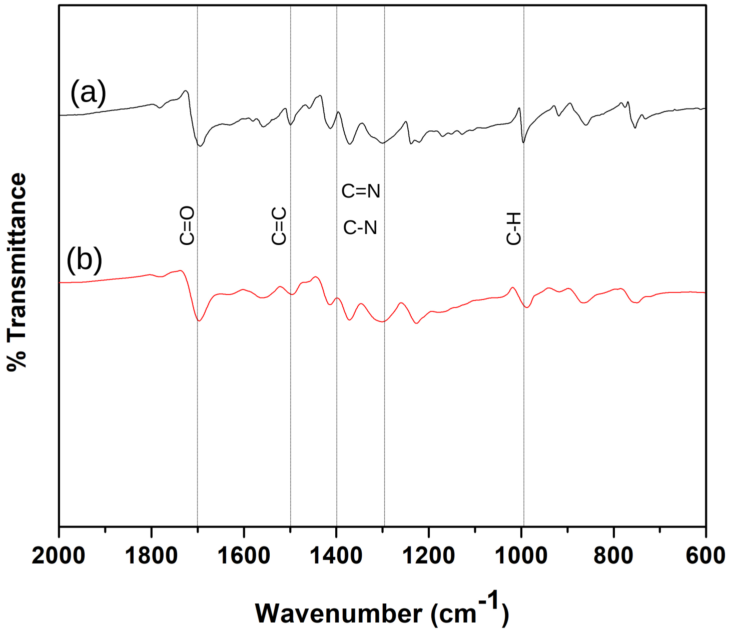

Figure S4. FTIR Spectra for neutral BBL film (a) before, and (b) after gas adsorption.

S3 Adsorption Energy

We have compared the DFT-calculated adsorption energy (E_{ads}) of BBL and the gas molecule with the pairwise interaction energy between BBL and the gas molecule calculated from MD simulations. The MD calculated E_{ads} includes both van der Waals and Coulombic interaction energies and we divided the E_{ads} by the number of total gas molecules to find the pairwise interaction energy per gas molecule.

S3.1 DFT Calculated Adsorption Energy and the Preferential Sites

We placed the gas molecules at different adsorption sites on a BBL chain and optimized the geometries, which are summarized in Table S3 and Table S4. Regardless of the initial position of the gas molecules, they tend to get adsorbed on the preferential sites. We found, the imidazole ring is the preferable adsorption site for H_2S molecule, see Table S3 and the pyridine ring is the preferable adsorption site for the NH_3 molecule, see Table S4.

Table S3. Geometries of H_2S molecule placed on different adsorption sites of BBL polymer before DFT geometry optimization (left) and after DFT geometry optimization (right), along with the corresponding adsorption energies.

Initial Position of H_2S Gas Molecule	E_{ads} (Initial) (eV)	Final Position of H_2S Gas Molecule	E_{ads} (final) (eV)
Benzene 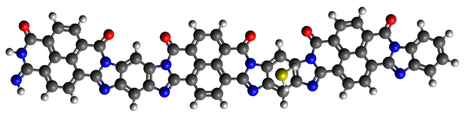	-0.1508	Imidazole 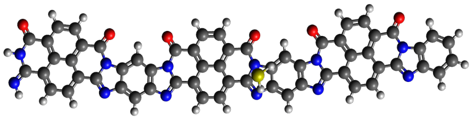	-0.2200
Naphthalene 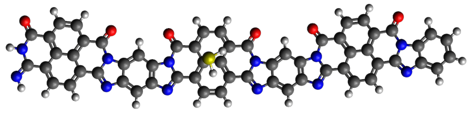	-0.1537	Naphthalene 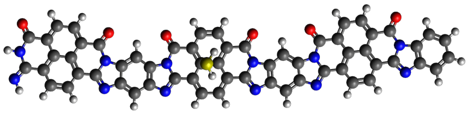	-0.1675
Pyridine 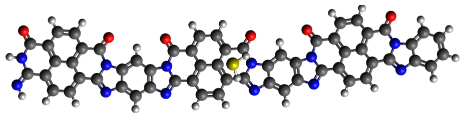	-0.1625	Imidazole 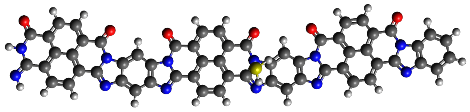	-0.2200
Imidazole 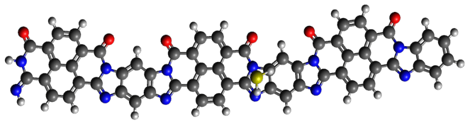	-0.2119	Imidazole 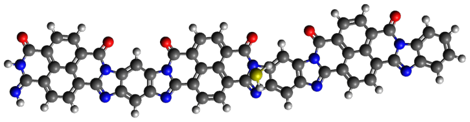	-0.2119

Table S4. Geometries of NH_3 molecule placed on different adsorption sites before DFT geometry optimization (left) and after DFT geometry optimization (right) along with the corresponding adsorption energies.

Initial Position of NH_3 Gas Molecule	E_{ads} (Initial) (eV)	Final Position of NH_3 Gas Molecule	E_{ads} (final) (eV)
Benzene 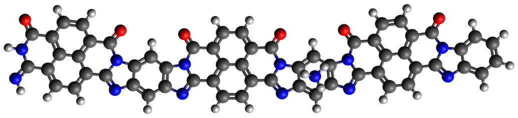	-0.1373	Pyridine 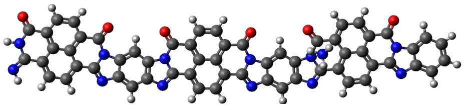	-0.3108
Naphthalene 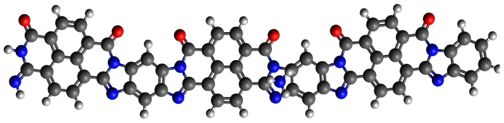	-0.1719	Pyridine 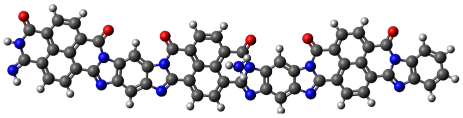	-0.3078
Pyridine 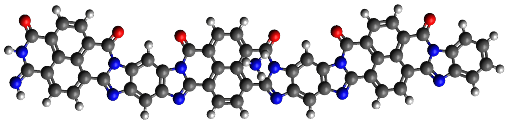	-0.3168	Pyridine 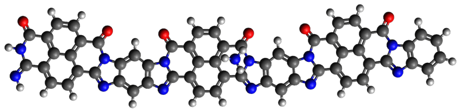	-0.3168
Imidazole 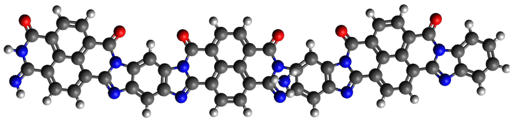	-0.1818	Pyridine 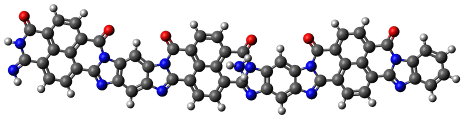	-0.3168

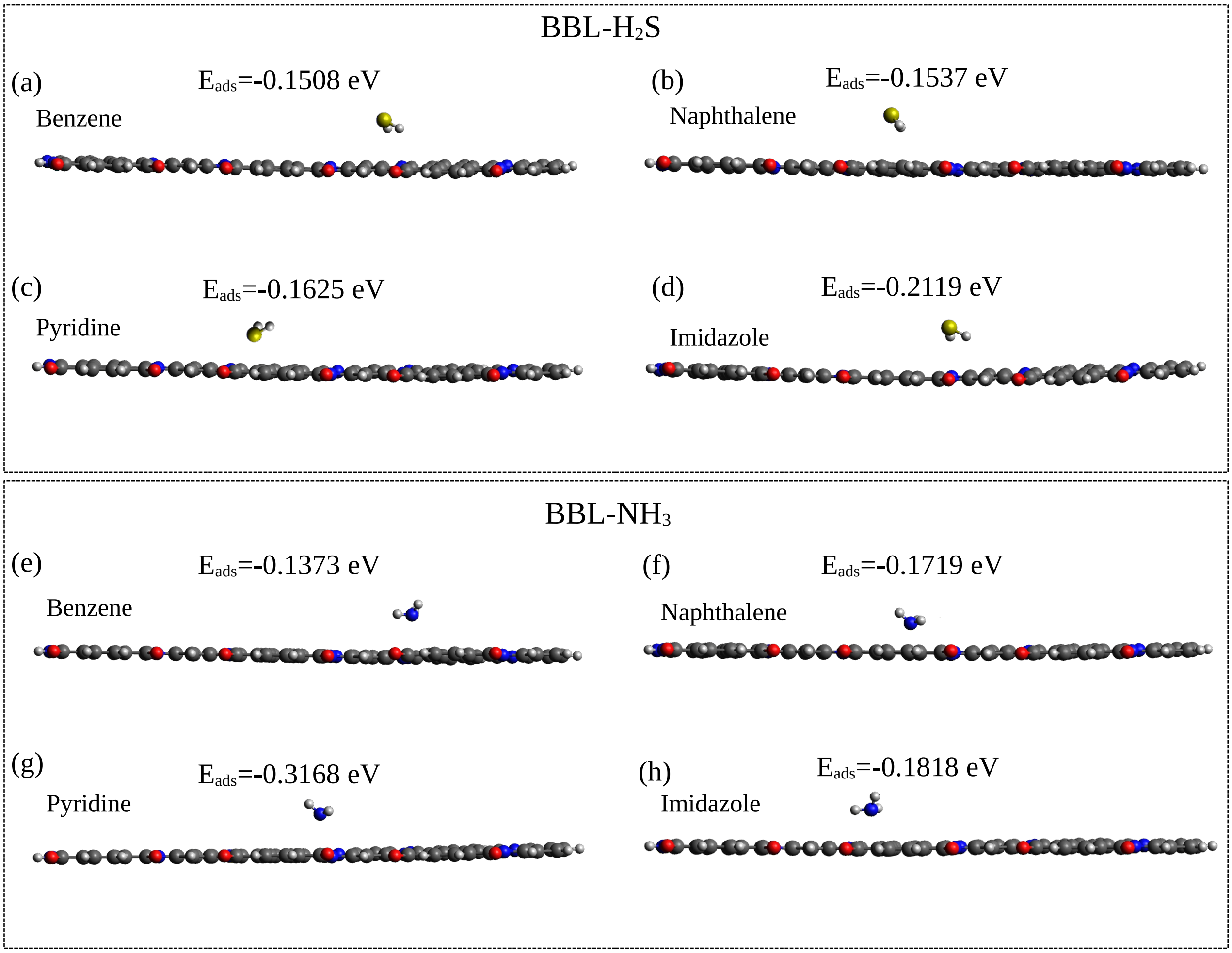

Figure S5. [Upper Panel] (a-d) The corresponding adsorption energies (E_{ads}) of one H_2S molecule when adsorbed on four different adsorption sites on top of a BBL trimer, namely, benzene, naphthalene, pyridine, imidazole, respectively, along with the geometries. [Lower Panel] (e-h) The corresponding E_{ads} of one NH_3 molecule when adsorbed on four different adsorption sites on a BBL trimer, namely, benzene, naphthalene, pyridine, imidazole, respectively, along with the respective geometries (side view).

S3.1.1 Gas Adsorption on the Top of a Single BBL Chain vs. $\pi-\pi$ Stacked Chains

Next, we compared the interaction of the two gas molecules, H_2S and NH_3, with a $\pi-\pi$-stack made of two BBL chains with the interaction of a single BBL chain, shown in Table S5. The strength of interaction of both the gas molecules with the two $\pi-\pi$ stacked BBL chains is slightly higher than that of the single BBL chain but the difference is very insignificant, which is attributed to the fact that the gas molecules are being adsorbed on the surface of the chain. We note from the bulk adsorption study from the MD simulations that gas adsorption is not affecting the $\pi-\pi$ stacked arrangements of the polymer chains.

Table S5. Calculated adsorption energy (E_{ads} (eV)) with the corresponding inter-molecular equilibrium distances (*d*) of H_2S and NH_3 for a single BBL chain and for two $\pi-\pi$ stacked BBL chains. $d_{N/S}$ (Å) represents the equilibrium distances of the Sulphur/Nitrogen atoms of H_2S/NH_3 from the center of mass of the adsorption site, whereas d (Å) represents the equilibrium distance calculated from the center of mass of the gas molecules to adsorption site in the BBL chain.

Analyte Gas	Optimized Geometry	E_{ads} (eV)	$d_{N/S}$ (Å)	d (Å)	E_g (eV)	CT
H_2S	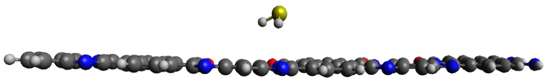	-0.21	3.728	3.42	5.80	0.0004
	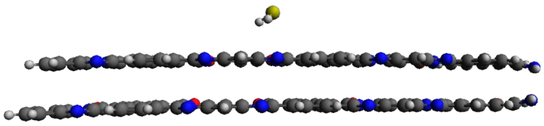	-0.22	3.748	3.46	5.71	0.0009
NH_3	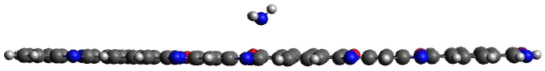	-0.32	3.24	3.58	5.75	0.0132
	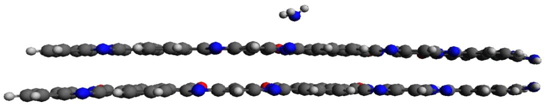	-0.33	3.236	3.48	5.65	0.0146

We analyzed the interaction energies and the equilibrium distances where the H_2S and the NH_3 gas molecules adsorbed at the preferential adsorption site of a single BBL polymer and of two $\pi-\pi$ stacked BBL polymers, see Table S5. E_{ads} is lower for NH_3 than H_2S which indicates the higher strength of interaction between NH_3 and the polymer chain. The calculated intermolecular equilibrium distance, d (Å), which represents the equilibrium distance calculated from the center of mass of the gas molecules to the adsorption site in the BBL chain, is lower for the case of H_2S than NH_3. This is attributed to the two hydrogen atoms of H_2S, which are closer to the BBL chain than the sulfur atom. We calculated the equilibrium distances of the Sulphur/Nitrogen atoms of H_2S/NH_3 from the center of mass of the adsorption site and found lower $d_{N/S}$ for NH_3 than H_2S. Hence, the orientation of the adsorbed H_2S and NH_3 molecules with respect to the BBL chain indicates that the nitrogen atoms of NH_3 are closer to the BBL than the hydrogen atoms of NH_3, whereas, hydrogen atoms of H_2S are closer than the sulfur atom of H_2S, resulting higher interaction energy and higher magnitude of charge transfer for BBL-NH_3 than that of BBL-H_2S.

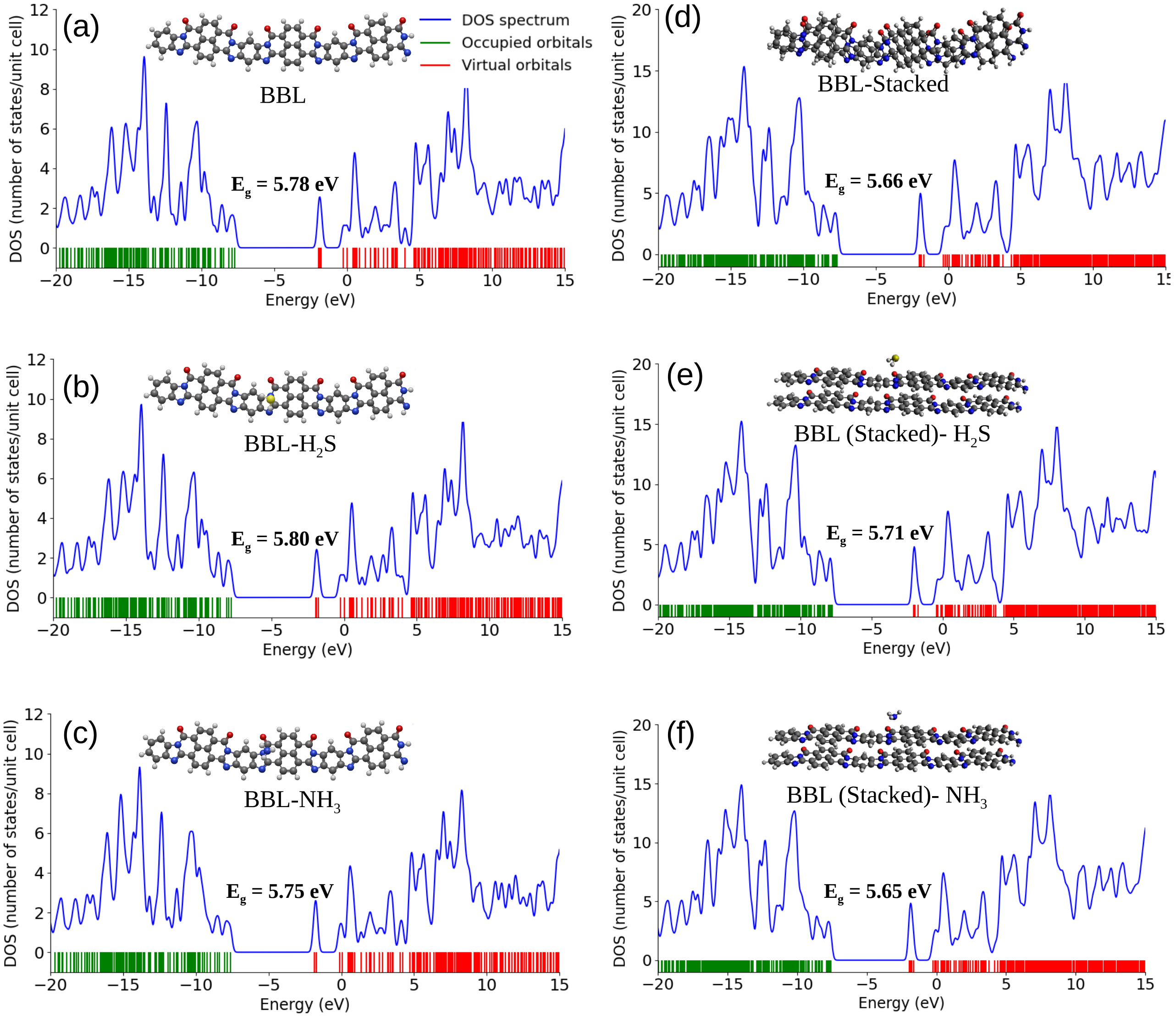

Figure S6. [Left panel] Density of States (DOS) along with the optimized geometry of (a) isolated BBL chain, (b) BBL-H_2S complex, and (c) BBL-NH_3 complex. [Right panel] Density of States (DOS) along with the optimized geometry of (d) stacked BBL chains, (e) BBL (stacked)-H_2S complex, and (f) BBL (stacked)-NH_3 complex.

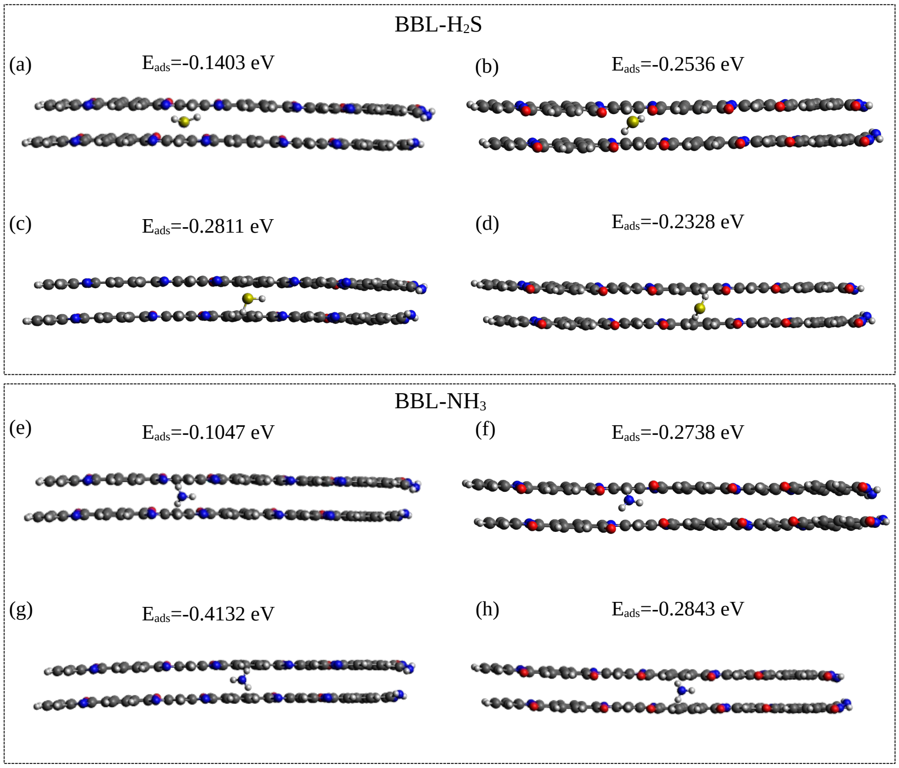

Figure S7. [Upper Panel] (a-d) The corresponding adsorption energies (E_{ads}) of one H_2S molecule placed on four different adsorption sites on the sides of stacked BBL chains, namely, close to oxygen-lean (E) and oxygen-rich (F) side of benzene and oxygen-rich (G) and oxygen-lean (H) side of naphthalene respectively, along with the geometries.[Lower Panel] (e-h) The corresponding E_{ads} of one NH_3 molecule placed on four different adsorption sites on the sides of stacked BBL chains, namely, close to oxygen-lean (E) and oxygen-rich (F) side of benzene and oxygen-rich (G) and oxygen-lean (H) side of naphthalene respectively, along with the geometries (side view).

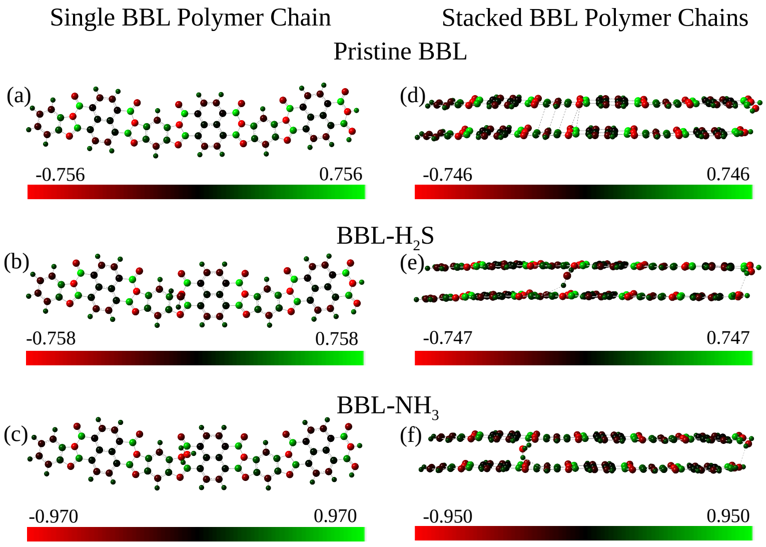

Figure S8. Charge distribution of (a) isolated BBL chain, (b) BBL-H_2S complex, (c) BBL- NH_3 complex, (d) stacked BBL chains, (e) BBL (stacked)- H_2S complex, and (f) BBL (stacked)- NH_3 complex.

S3.2 Interaction with Other Gases

For both BBL-H_2S and BBL-NH_3 systems, the preferred adsorption site was found to be the site near an imidazole ring and a pyridine ring. We placed other common gases present in the air, such as O_2, N_2, CO_2, and H_2O, in between an imidazole ring and a pyridine ring and relaxed the geometries using DFT without imposing any constraints. The optimized geometries are shown in Figure S9 and $|E_{ads}|$, interaction distance, and the magnitude of charge transfer for all the gases are shown in Figure S10.

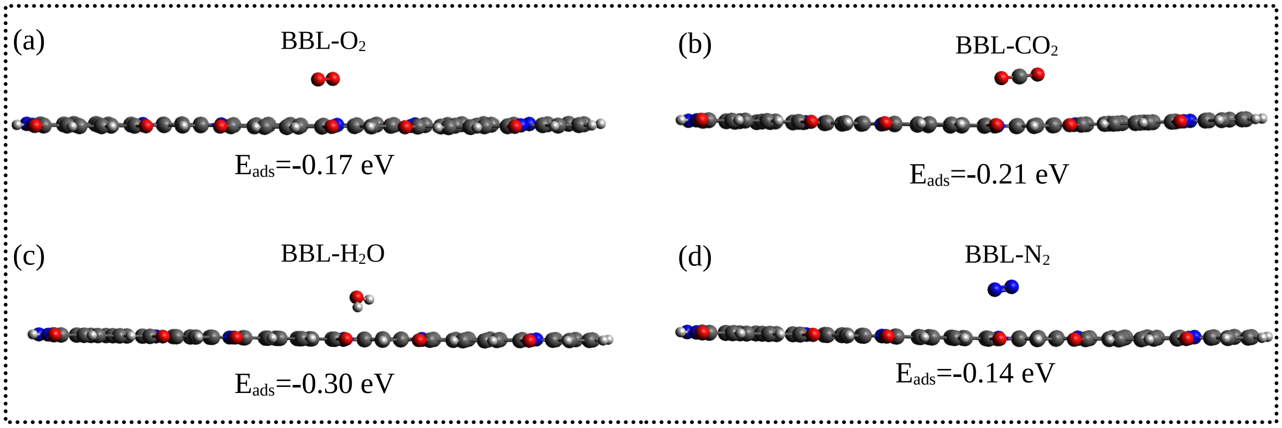

Figure S9. DFT optimized geometry of various other gas molecules on the BBL chain along with the corresponding adsorption energies (E_{ads}(eV)).

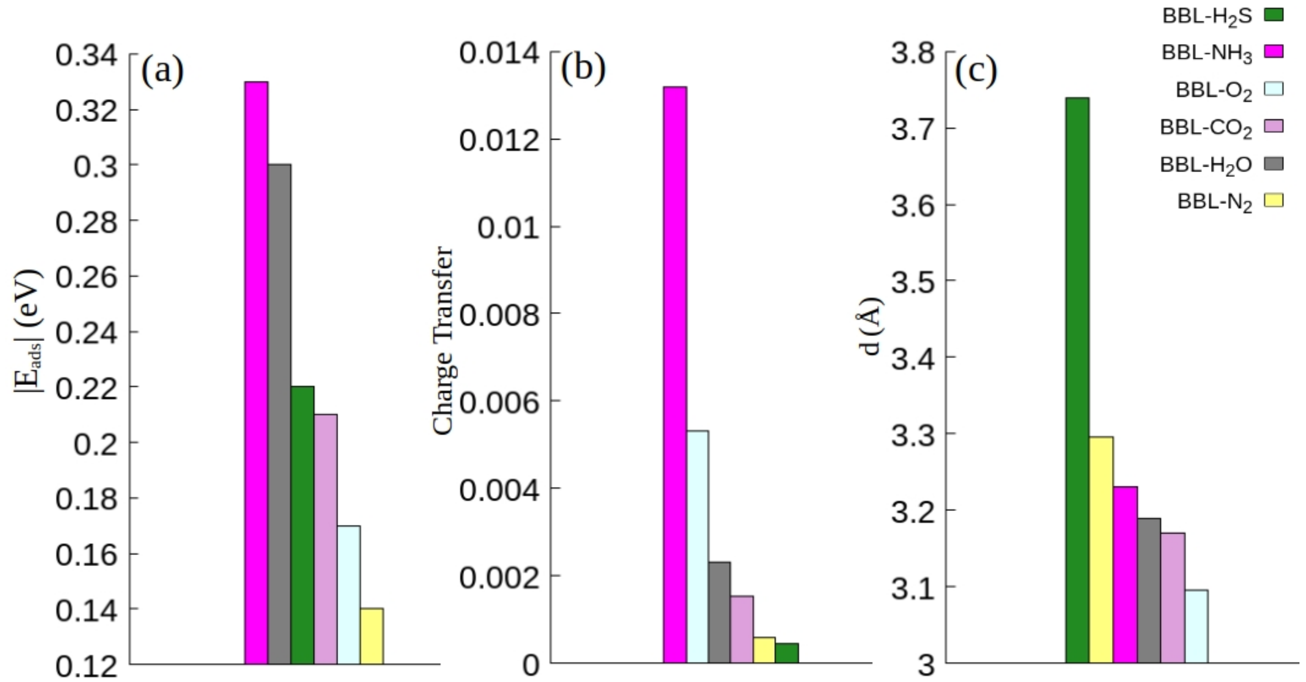

Figure S10. The comparison of (a) adsorption energy (eV), (b) magnitude of charge transfer, and (c) equilibrium distance, d (Å) of BBL-H_2S and BBL-NH_3 with various other gas molecules.

S3.3 Electronic Structures

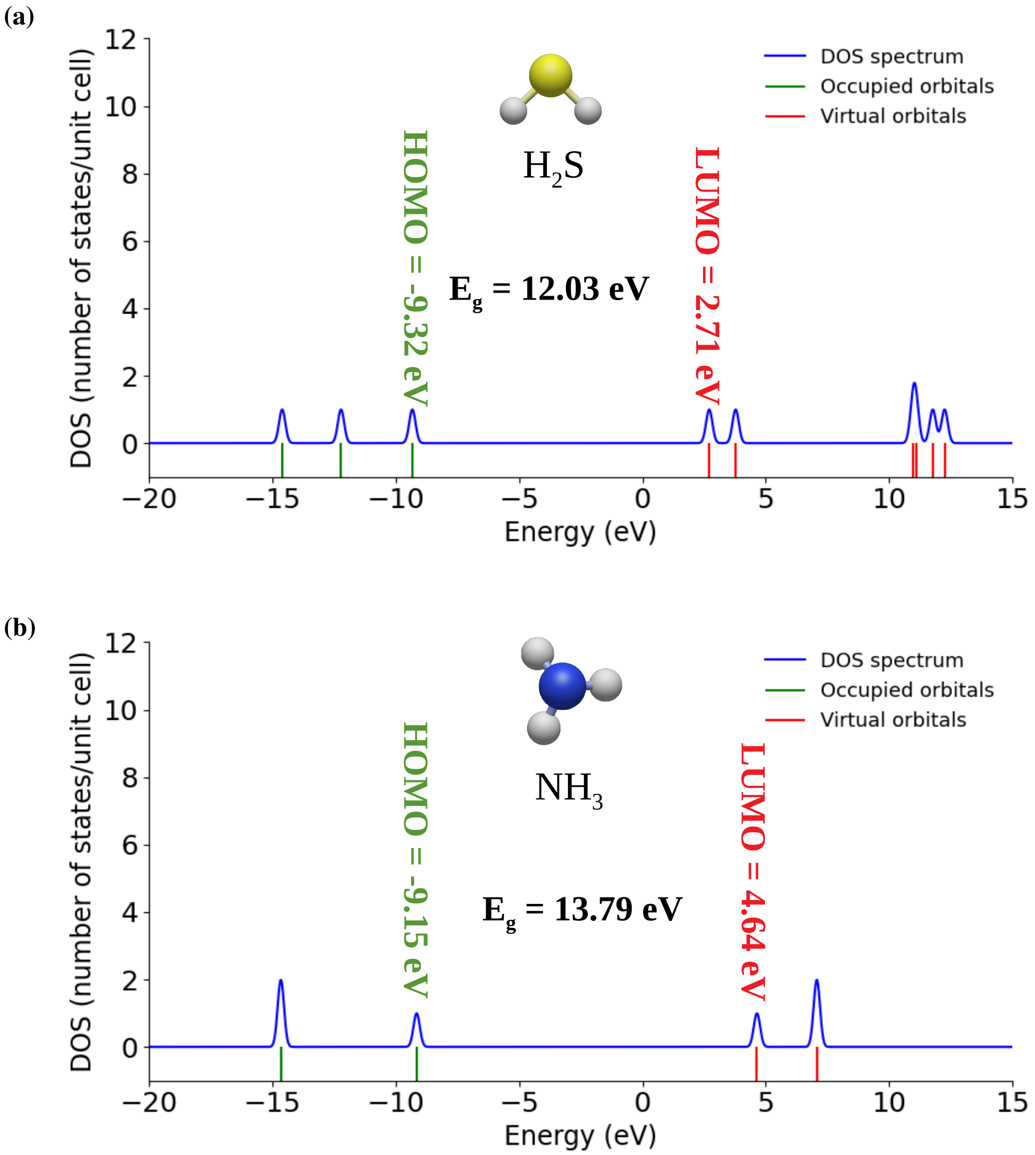

Figure S11. Density of states (DOS) of isolated (a) H_2S, and (b) NH_3 molecule. The corresponding HOMO, LUMO, and the HOMO-LUMO bandgap (E*g*) are marked within the figure.

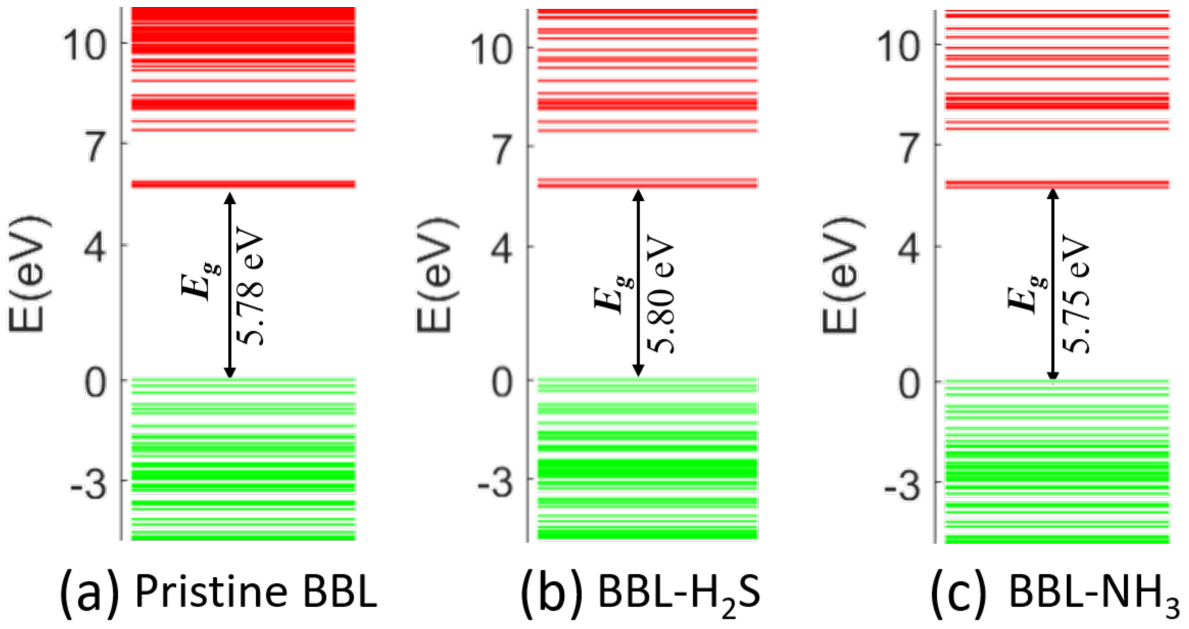

Figure S12. Molecular orbital diagrams of (a) Pristine BBL, (b) BBL-H_2S, and (c) BBL-NH_3. The energy lines in the valence band and the conduction band are shown in green and red, respectively. The corresponding energy gap (E_g) between the valence and conduction band of all three systems are marked.

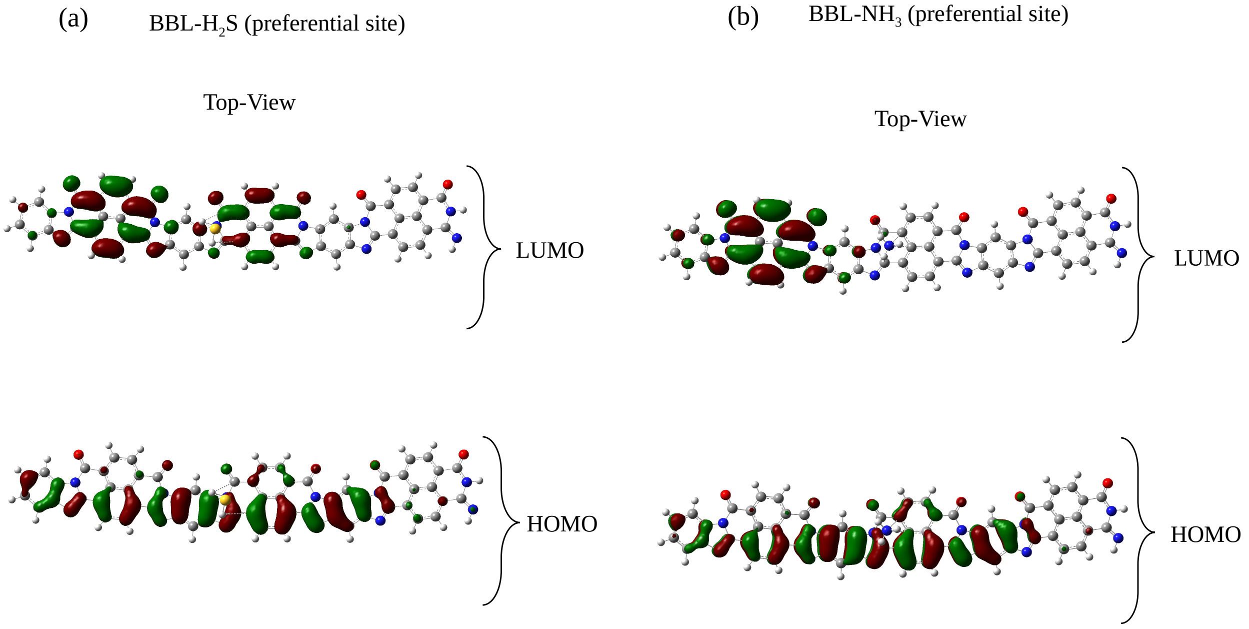

Figure S13. Molecular orbital distribution of HOMO- LUMO levels for preferential sites for (a) BBL-H_2S and (b) BBL-NH_3.

S3.4 UV-vis Absorption Spectra

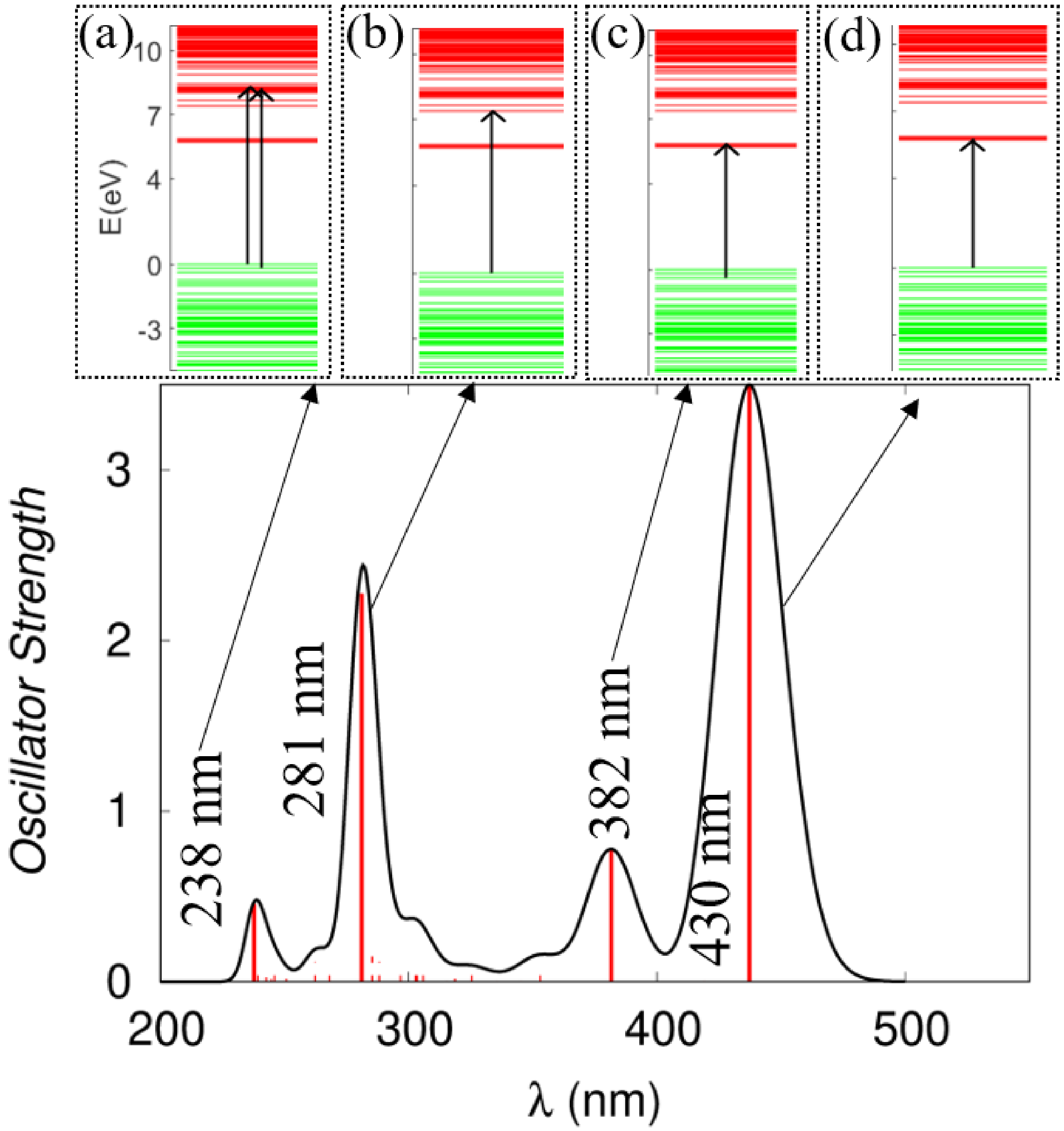

Figure S14. The corresponding electronic transitions of all four peaks present in the UV-vis absorption spectra of Pristine BBL. (a,b) The two peaks in the lower wavelength are due to the electronic transitions from HOMO and HOMO-1 to the higher-lying unoccupied energy levels attributed to the mini-band formation in the conduction band. (c) The peak at 382 nm is due to the electronic transition from HOMO-3 to the LUMO. (d) The peak at the highest wavelength (430nm) is due to the HOMO-LUMO electronic transition.

Figure S15(a-e) shows the UV-vis absorption spectra of an isolated BBL chain and spectra of BBL-H_2S, and BBL-NH_3 complexes, where the H_2S and NH_3 gases are adsorbed on the preferential adsorption site on top of an isolated BBL chain and Figure S15(f-j) shows the UV-vis absorption spectra of a $\pi - \pi$ stack and spectra of BBL-H_2S, and BBL-NH_3 complexes, where the H_2S and NH_3 gases are adsorbed on the preferential adsorption site on the side of a $\pi - \pi$ stack. Let us discuss the evolution of UV-vis absorption spectra of the BBL-H_2S and BBL-NH_3 systems when the gas molecules are adsorbed on the top-site of BBL, shown in Figure S15(a-e). The UV-vis absorption spectra of isolated BBL show two distinct electronic transitions resulting in two peaks at the lower wavelengths, viz. P1≈238 nm and P2≈281 nm, and two distinct electronic transitions resulting in two peaks at the higher wavelength, viz. P3≈382 nm and P4≈430 nm, see Figure S15(f). The peaks in the

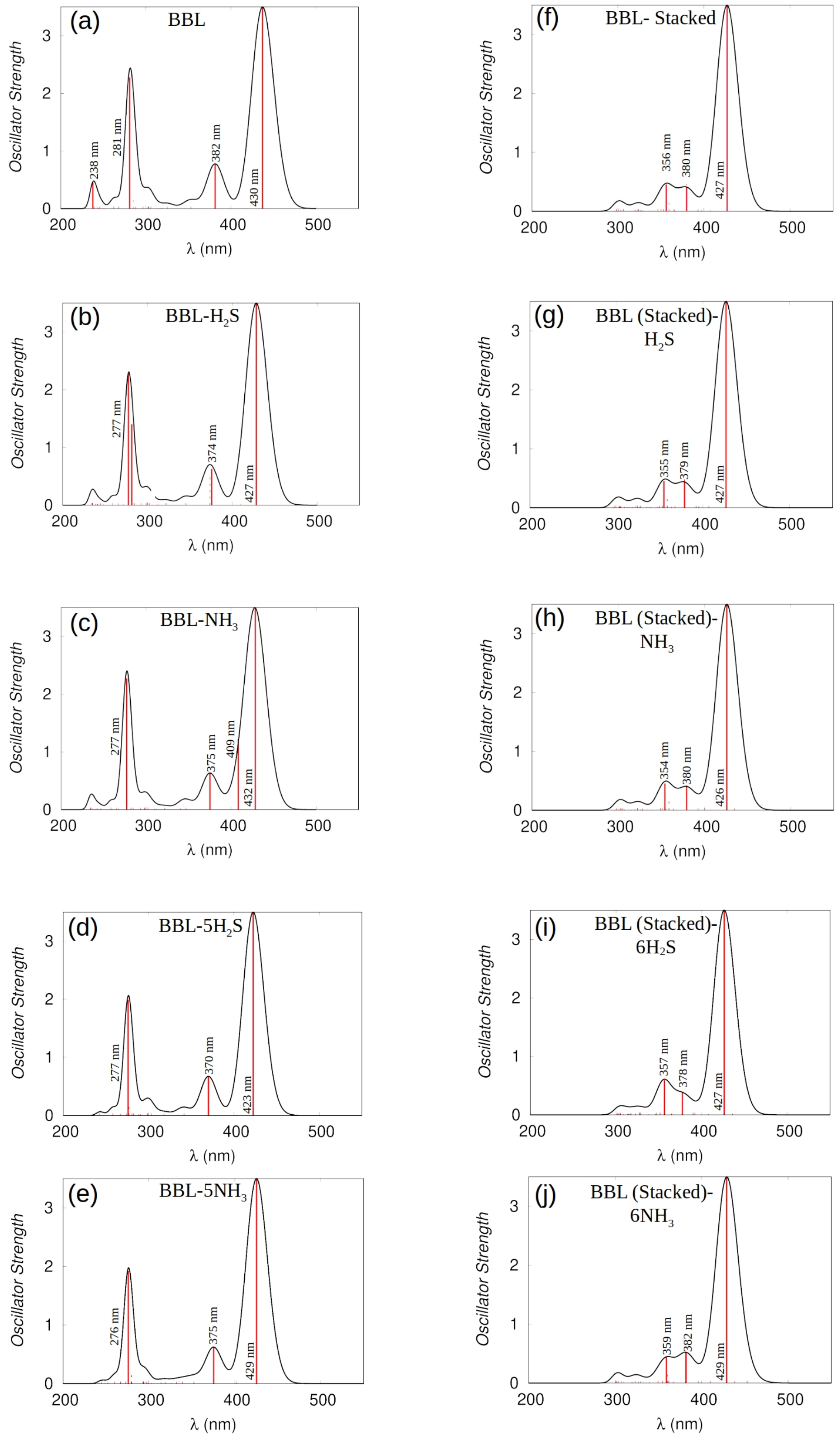

Figure S15. [Left panel] UV-vis absorption spectra of (a) isolated BBL chain, (b) BBL-H_2S complex, (c) BBL-NH_3 complex, (d) BBL-5H_2S complex, and (e) BBL-5NH_3 complex. [Right panel] UV-vis absorption spectra of (f) stacked BBL chains, (g) BBL (stacked)-H_2S complex, (h) BBL (stacked)-NH_3 complex, (i) BBL (stacked)-6H_2S complex, and (j) BBL (stacked)-6NH_3 complex.

absorption spectra result from the electronic transitions from the occupied energy levels of the valence band to the unoccupied

Table S6. Oscillator strengths of all the peaks in the UV-vis absorption spectra shown in Figure 7 and S15.

System	Peak Wavelength (nm)	Oscillator Strength
BBL	$P_{1.}$ 238 nm<br>$P_{2.}$ 281 nm<br>$P_{3.}$ 382 nm<br>$P_{4.}$ 430 nm	1. 0.2329<br>2. 1.5016<br>3. 0.5593<br>4. 2.5898
BBL-H_2S	$P_{1.}$ 239 nm<br>$P_{2.}$ 277 nm<br>$P_{3.}$ 374 nm<br>$P_{4.}$ 427 nm	1. 0.0213 (not considered)<br>2. 1.5016<br>3. 0.5250<br>4. 2.6087
BBL-5H_2S	$P_{1.}$ Absent<br>$P_{2.}$ 277 nm<br>$P_{3.}$ 370 nm<br>$P_{4.}$ 423 nm	1. Absent<br>2. 1.4936<br>3. 0.5286<br>4. 2.6152
BBL-NH_3	$P_{1.}$ 237 nm<br>$P_{2.}$ 277 nm<br>$P_{3.}$ 375 nm<br>$P_{4.}$ 432 nm	1. 0.0290 (not considered)<br>2. 1.6308<br>3. 0.4569<br>4. 2.4721
BBL-5NH_3	$P_{1.}$ Absent<br>$P_{2.}$ 276 nm<br>$P_{3.}$ 375 nm<br>$P_{4.}$ 429 nm	1. Absent<br>2. 1.6334<br>3. 0.4594<br>4. 0.2017

energy levels of the conduction band, shown in Supplementary Fig. S14, and the oscillator strengths of the electronic transitions are shown in Supplementary Table S6. The peak at the highest wavelength (at ∼430 nm) is due to the electronic transition from the HOMO to the LUMO, and the peaks at the lower wavelengths result from the lower-lying occupied energy levels to the higher-lying unoccupied energy levels, see Supplementary Fig. S14. Upon interaction with both the gas molecules, H_2S and NH_3, the first peak at the lower wavelength (P1), ∼ 238 nm, present in the absorption spectra of pure BBL as shown in Figure S15(a), almost diminishes in Figure S15(b,c) as the oscillator strengths of P1 decreases significantly. This is more prominent in the case of multiple adsorbed gas molecules, see Figure S15(d,e). The second peak at the lower wavelength (P2), ∼281 nm, present in the absorption spectra of pure BBL as shown in Figure S15(a), shows a blue shift to ∼277 nm, see Figure S15(b), and (c). The first peak at the higher wavelength (P3) also shows a blue shift from ∼382 nm to ∼374 nm for BBL-H_2S complex and to ∼375 nm for BBL- NH_3 complex. The second peak of the higher wavelength (P4) of the absorption spectra of only the BBL chain at ∼430 nm, which represents the HOMO to LUMO electronic transition, upon interaction with H_2S and NH_3 ranges from 423-429 nm. However, the UV-vis absorption spectra for the case of gas molecules adsorbed on the side-site of BBL remain almost the same for both BBL-H_2S and BBL-NH_3 systems as shown in Figure S15(f,j).

S4 Simulation Details

Berny algorithm, a quasi-Newton method(5), was used in geometry optimization during all DFT calculations. During geometry optimization, the force convergence threshold value was set to $\sim 1 \times 10^{-2}$eV/Å and threshold value of the average displacement was set to $\sim 6.3 \times 10^{-4}$Å. The SCF energy convergence threshold values for the density matrix was set to $\sim 2\times10^{-7}$eV. Note, as implemented in the Gaussian code, the energy threshold value is not defined as a convergence criterion in SCF calculation; however, the threshold value of the density matrix of $\sim 2\times10^{-7}$eV corresponds to energy threshold value of $\sim 2\times10^{-14}$eV.

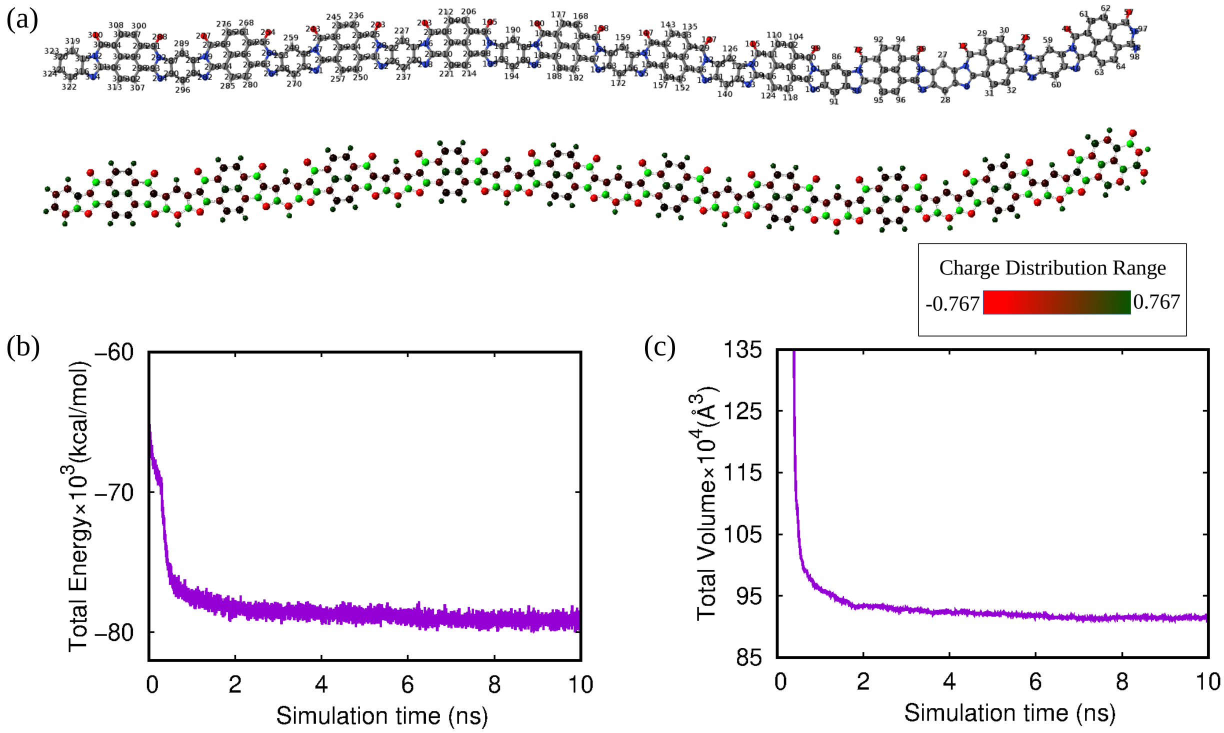

Figure S16. (a) Charge distribution image of the BBL chain, (b) Energy vs. simulation time, and (c) volume of the simulation box vs. simulation time.

All the MD simulations were performed in an NPT ensemble with Nose—Hoover barostat and thermostat and at a timestep of 2 fs using the LAMMPS simulation package. To prepare the dry films, we first annealed the film at 503 K and then quenched it to room temperature (303 K) at a cooling rate of 4 $K/100ps$. We equilibrated the system at 303 K and 1 atm for $\sim$10 ns. The Lennard-Jones interaction cutoff was set to 0.9 nm. Furthermore, the K-scheme of the particle-particle-particle-mesh(6) method was used to calculate the long-range Coulombic interaction, using a 1.0 nm cutoff. We used the general AMBER force field (GAFF) to describe the bonded and non-bonded interactions among the atoms generated using the Moltemplate code(7). In each state, we calculated the corresponding partial charges using DFT with the functional, ωb97xd and the basis set, 6-31g(d) in the Gaussian package. The charge distribution of the neutral BBL chain is shown in fig. S16.

To simulate the BBL-H_2S and BBL-NH_3 systems, we first placed the dry film of BBL in the center of the simulation box and then surrounded it with 2000 gas molecules of either H_2S or NH_3 in a computational box, see Fig.9. The system was then equilibrated in an isothermal-isobaric (NPT) ensemble at a temperature of 303 K and a pressure of 1 atm for 10 ns. To ensure the system is equilibrated, we plotted the total energy vs. simulation time and the total volume vs. simulation time, as shown in fig. S16.

References

1. Coleman, S., Spearot, D. & Capolungo, L. Virtual diffraction analysis of ni [0 1 0] symmetric tilt grain boundaries. *Model. Simul. Mater. Sci. Eng.* **21**, 055020 (2013).

2. Yao, Z.-F., Wang, J.-Y. & Pei, J. Control of π–π stacking via crystal engineering in organic conjugated small molecule crystals. *Cryst. Growth & Des.* **18**, 7–15 (2018).

3. Sharma, A., Malani, A., Medhekar, N. V. & Babarao, R. Co 2 adsorption and separation in covalent organic frameworks with interlayer slipping. *CrystEngComm* **19**, 6950–6963 (2017).

4. Wang, S. *et al.* Sequential doping of ladder-type conjugated polymers for thermally stable n-type organic conductors. *ACS Appl. Mater. & Interfaces* **12**, 53003–53011 (2020).

5. Schlegel, H. B. Optimization of equilibrium geometries and transition structures. *J. computational chemistry* **3**, 214–218 (1982).

6. Hockney, R. & Eastwood, J. Computer simulation using particles taylor & francis. *Inc., USA* (1988).

7. Jewett, A. I. *et al.* Moltemplate: A tool for coarse-grained modeling of complex biological matter and soft condensed matter physics. *J. Mol. Biol.* **433**, 166841, DOI: https://doi.org/10.1016/j.jmb.2021.166841 (2021). Computation Resources for Molecular Biology.
